# Keratinocyte TMEM79 protects early life epidermal homeostasis and restrains STING-dependent skin inflammation

**DOI:** 10.1093/pnasnexus/pgag361

**Published:** 2026-10-28

**Authors:** Mengxu Ge, Danilo Predes, Andrew C Kwong, Eunice To, Maorong Chen, Aakanksha Jain, Wenyi Wei, Jose Ordovas-Montanes, Juan-Manuel Leyva-Castillo, Isaac M Chiu, Xi He

**Affiliations:** F. M. Kirby Neurobiology Center, Boston Children's Hospital, Harvard Medical School, Boston, MA 02115, USA; F. M. Kirby Neurobiology Center, Boston Children's Hospital, Harvard Medical School, Boston, MA 02115, USA; Division of Gastroenterology, Hepatology, and Nutrition, Boston Children's Hospital, Boston, MA 02115, USA; Harvard Graduate Program in Biological and Biomedical Sciences, Harvard Medical School, Boston, MA 02115, USA; Broad Institute of MIT and Harvard, Cambridge, MA 02142, USA; F. M. Kirby Neurobiology Center, Boston Children's Hospital, Harvard Medical School, Boston, MA 02115, USA; F. M. Kirby Neurobiology Center, Boston Children's Hospital, Harvard Medical School, Boston, MA 02115, USA; F. M. Kirby Neurobiology Center, Boston Children's Hospital, Harvard Medical School, Boston, MA 02115, USA; Department of Immunology, University of Washington, Seattle, WA 98109, USA; Department of Pathology, Beth Israel Deaconess Medical Center, Harvard Medical School, Boston, MA 02215, USA; Division of Gastroenterology, Hepatology, and Nutrition, Boston Children's Hospital, Boston, MA 02115, USA; Broad Institute of MIT and Harvard, Cambridge, MA 02142, USA; Program in Immunology, Harvard Medical School, Boston, MA 02115, USA; Harvard Stem Cell Institute, Cambridge, MA 02138, USA; Ragon Institute of MGH, MIT and Harvard, Cambridge, MA 02139, USA; Division of Immunology, Boston Children's Hospital, Harvard Medical School, Boston, MA 02115, USA; Department of Immunology, Harvard Medical School, Boston, MA 02115, USA; Gene Lay Institute of Immunology and Inflammation of Brigham and Women's Hospital, MGH, and Harvard Medical School, Boston, MA 02115, USA; F. M. Kirby Neurobiology Center, Boston Children's Hospital, Harvard Medical School, Boston, MA 02115, USA

**Keywords:** TMEM79, keratinocytes, ferroptosis, STING, epidermis, type 17 immunity

## Abstract

Chronic inflammatory skin diseases are characterized by epithelial barrier dysfunction and immune dysregulation, yet the epithelial-intrinsic mechanisms that initiate inflammation remain poorly understood. TMEM79 is a dermatitis-associated transmembrane protein expressed in keratinocytes, but how its loss promotes skin disease remains unclear. Here, using keratinocyte-specific *Tmem79* conditional knockout (cKO) mice and TMEM79-deficient human keratinocytes, we find that TMEM79 is required for proper epithelial differentiation and barrier formation, and that its loss induces keratinocyte stress and ferroptosis-associated lipid peroxidation. Longitudinal analysis of neonatal and early postnatal *Tmem79*-deficient skin revealed disrupted epidermal homeostasis and early type I IFN signaling preceding the later emergence of type 17 inflammation. Mechanistically, genetic ablation of *Sting1*, but not *Mavs*, ameliorated dermatitis and downstream type 17 inflammation, while IL-17A was required for subsequent epidermal hyperplasia. These findings establish STING-dependent signaling as an upstream driver of skin immune activation and pathology. Together, our findings link early-life epithelial stress to innate immune activation and suggest that targeting lipid peroxidation or STING signaling may help prevent inflammatory skin disease progression.

Significance Statement Inflammatory skin diseases such as atopic dermatitis and psoriasis are among the most prevalent chronic inflammatory disorders. While barrier disruption is a hallmark of these diseases, the processes that initiate this dysfunction in epithelial cells and promote downstream immune responses remain poorly understood. Using conditional knockout mice and human keratinocytes, we show that keratinocyte-specific loss of TMEM79, a dermatitis-associated protein, causes early defects in epithelial differentiation and barrier formation, accompanied by ferroptosis-associated lipid peroxidation. In mice, TMEM79 deficiency induces STING-dependent type I IFN signaling and downstream type 17 immune responses that drive epithelial hyperplasia and skin pathology. Our findings reveal how early-life keratinocyte dysfunction can promote sustained inflammation and identify potential therapeutic targets to prevent disease progression.

## Introduction

Inflammatory skin disorders such as atopic dermatitis (AD) and psoriasis are among the most prevalent chronic inflammatory diseases and represent a major public health burden (1, 2). They are characterized by epidermal barrier dysfunction, immune cell infiltration, and recurrent inflammation driven by genetic and environmental interactions. Although traditionally viewed as primarily immune-mediated skin conditions, increasing evidence indicates that epidermal dysfunction serves as an initiating event. Notably, genetic and functional studies in both AD and psoriasis have identified keratinocyte-intrinsic defects in barrier function and stress responses as important initiators of cutaneous inflammation (3–9).

Keratinocytes are the most abundant cell type in the epidermis and play dual roles in barrier formation and immune surveillance. Through terminal differentiation, they generate the stratum corneum (SC), which forms a physical and biochemical barrier that limits water loss and prevents entry of pathogens and allergens (10). In parallel, keratinocytes act as immune sentinels that respond to microbial, environmental, and tissue-derived signals by secreting cytokines, chemokines, and antimicrobial peptides to shape local inflammatory responses (11, 12). Disruption of these tightly coordinated processes compromises epidermal homeostasis, resulting in abnormal differentiation, cellular stress, and impaired barrier integrity. Specialized programed forms of keratinocyte cell death, such as cornification, are required for normal barrier formation, whereas dysregulated or stress-induced cell death can disrupt barrier integrity and trigger inflammation through the release of alarmins and danger-associated molecular patterns (DAMPs) that activate innate immune sensing pathways (13–15).

Among proteins implicated in epidermal homeostasis and barrier function, TMEM79 (also known as Mattrin) has emerged as an intriguing yet incompletely characterized regulator. TMEM79 is a five-pass transmembrane protein predominantly expressed in keratinocytes (16, 17), and genetic variants are associated with human AD (17). Genetic studies in flaky-tail mice first demonstrated that the *matted* allele, a spontaneous loss of function mutation in *Tmem79*, was responsible for the dermatitis and heightened allergen hypersensitivity observed in these mice (16, 17). Subsequent work showed that global *Tmem79*-deficient mice developed barrier dysfunction, epidermal hyperplasia, Th17 immune responses, mast cell accumulation, microbial dysbiosis, and itch behaviors resembling human disease (18–21). Cell-specific studies further showed that TMEM79 function in both keratinocytes and sensory neurons contributed to itch and scratching behaviors (20). *Tmem79*-deficient mice developed dermatitis in a biphasic manner: An early phase arises at 2–6 weeks of age that occurs independently of adaptive immunity and the skin microbiota, followed by a chronic phase driven by skin microbial dysbiosis and type 17 immune responses (18, 19). Genetic ablation of adaptive immune cells, *Il-17a*, or antibiotic treatment markedly attenuates the chronic phase with minimal impact on early disease, indicating that the initial phase is driven by epithelial-intrinsic mechanisms. However, the keratinocyte changes and signals that govern this early phase and enable progression to the chronic type 17 inflammation remain undefined. Defining these mechanisms may reveal therapeutic targets during a critical early-life window that determines progression to chronic inflammation.

TMEM79 has been linked functionally to multiple aspects of epidermal biology, playing roles in lamellar granule (LG) secretion and SC formation (16). It shares distant sequence homology with membrane-associated proteins in eicosanoid and glutathione metabolism (MAPEG) family proteins, which are involved in lipid mediator metabolism and detoxification of reactive electrophiles (17, 20). TMEM79 has been shown to regulate oxidative stress and lipid homeostasis, and its loss is associated with increased sebaceous lipid production (17, 18, 20). TMEM79 has also been proposed to regulate temperature sensation by acting as a negative regulator of the thermosensitive ion channel TRPV3 (22). In addition, we recently identified TMEM79 as a specific antagonist of Wnt/β-catenin signaling that promotes the degradation of the Wnt receptor Frizzled (23).

Despite these advances, how TMEM79 deficiency initiates early-life dermatitis and licenses subsequent immune responses remain unclear. In this study, we utilized keratinocyte-specific *Tmem79* knockout mice and TMEM79-deficient human keratinocytes to show that TMEM79 mediates epidermal differentiation and barrier formation. This keratinocyte-intrinsic defect disrupts epidermal differentiation and barrier formation, is associated with ferroptotic lipid stress, and activates STING-dependent type I interferon responses during the initial phase of dermatitis. Over time, this pathology drives a type 17-dominated immune response, revealing how keratinocyte-intrinsic defects can disrupt epidermal homeostasis and initiate inflammatory skin disease.

## Results

### Spatial and temporal expression patterns of *Tmem79* in keratinocytes

To begin to define the role of TMEM79 in epidermal homeostasis, we first examined its spatial and age-dependent expression in the skin. Ear skin from wild-type (WT) mice was enzymatically separated into epidermal and dermal sheets (SI Appendix, Figure S1A). Enrichment of epidermal markers (*K14* and *K10*) in epidermal sheets and the dermal marker (*Col1a1*) in dermal sheets validated the effectiveness of separation (SI Appendix, Figure S1B). Quantitative PCR (qPCR) analysis showed that *Tmem79* was predominantly expressed in the epidermis (Figure 1A). To identify the cellular origin of this signal, we analyzed a published single-cell RNA-sequencing (scRNA-seq) dataset of mouse adult dorsal skin in telogen (24). *Tmem79* expression was enriched in *Dkkl1*+ suprabasal keratinocytes relative to basal and hair follicle-associated keratinocytes (SI Appendix, Figure S1C and Figure 1B).

**Figure 1:**
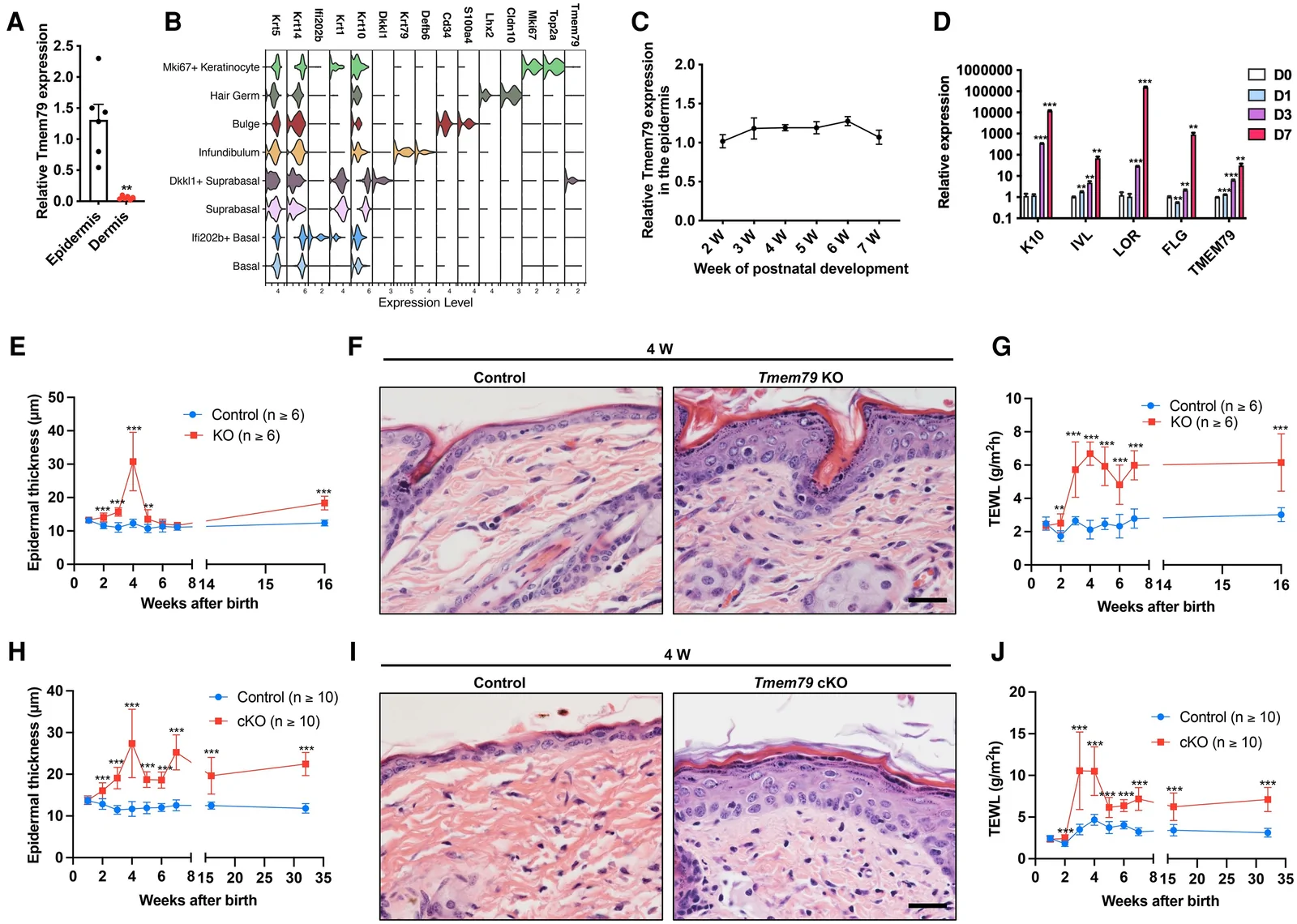
*Tmem79* deficiency in keratinocytes leads to epidermal hyperplasia and skin barrier dysfunction. A) qPCR analysis of *Tmem79* expression in the epidermis and dermis of 4-week-old WT mice. B) Violin plots showing expression of *Tmem79* and keratinocyte subtype markers across epidermal cell populations using a published scRNA-seq dataset from 9-week-old mice in the second telogen. C) qPCR analysis of *Tmem79* expression in the epidermis across developmental stages. W, weeks. D) qPCR analysis of differentiation markers and *Tmem79* expression during induced keratinocyte differentiation. D0 indicates the day of differentiation induction (day 0). The y-axis is shown on a log10 scale. E) Quantification of epidermal thickness over time in control and global KO mice. F) Hematoxylin and eosin (H&E) staining of dorsal skin from 4-week-old control and KO mice. Scale bar = 25 μm. G) Quantification of TEWL over time in control and KO mice. H) Quantification of epidermal thickness over time in control and cKO mice. I) H&E staining of dorsal skin from 4-week-old control and cKO mice. Scale bar = 25 μm. J) Quantification of TEWL over time in control and cKO mice. WT or heterozygous littermates served as controls for global KO mice, and *Tmem79 fl/fl* or K14-Cre; *Tmem79 fl/+* littermates served as controls for cKO mice. This control strategy was applied across all assays. Both male and female mice were used. For most assays, at least six biological replicates were used, unless otherwise stated. For comparisons between two groups, data are presented as mean ± SEM; *P < 0.05, **P < 0.01, ***P < 0.001 (unpaired two-tailed Student's *t*-test), unless otherwise stated.

To examine age-dependent changes in *Tmem79* expression from 2 weeks of age into adulthood, we performed qPCR analysis on WT epidermal sheets collected across developmental stages. *Tmem79* expression remained relatively stable between 2 and 7 weeks of age (Figure 1C). To complement these observations made in mice, we cultured primary human keratinocytes under conditions that either maintained a proliferative basal-like state or induced differentiation with high calcium over 1, 3, or 7 days (SI Appendix, Figure S1D). Progressive keratinocyte differentiation was confirmed by time-dependent enrichment of both early/intermediate (*K10* and *IVL*) and terminal differentiation markers (*LOR* and *FLG*) (Figure 1D). *TMEM79* expression levels increased concomitantly with this keratinocyte differentiation (Figure 1D). Consistent with these data, scRNA-seq data generated from human skin organoids showed enrichment *of TMEM79* in differentiated suprabasal keratinocytes (SI Appendix, Figure S1E) (25).

Overall, these results indicate that *TMEM79* is expressed primarily in suprabasal keratinocytes across human and mouse skin and shows stable expression during early postnatal development.

### Keratinocyte-specific *Tmem79* loss disrupts skin barrier homeostasis

To investigate the specific role of TMEM79 in keratinocytes, we compared global knockout (KO) and K14-Cre keratinocyte-specific conditional knockout (cKO) mice. qPCR analysis of whole-skin and epidermal samples confirmed complete deletion of *Tmem79* in whole skin from global KO mice and near-complete deletion in the epidermis of cKO mice, whereas low residual expression persisted in cKO whole skin (SI Appendix, Figure S2A and B), indicating low levels of *Tmem79* expression in nonkeratinocyte cell populations in the skin.

To assess the impact of *Tmem79* loss on skin structure and barrier function, we performed histological analysis of dorsal skin architecture and transepidermal water loss (TEWL) over time. Epidermal hyperplasia and barrier dysfunction as indicated by higher TEWL measurements were apparent as early as 2 weeks of age in KO mice, with the most pronounced changes observed at 4 weeks (Figure 1E–G). Notably, keratinocyte-specific deletion of *Tmem79* recapitulated the inflammatory skin phenotype observed in global KO mice (Figure 1H–J), indicating that pathology is primarily driven by loss of epithelial *Tmem79*.

Based on these findings, we focused subsequent analyses on two key timepoints: (1) 4 weeks of age, corresponding to peak pathology, and (2) earlier than 2 weeks of age to examine disease initiation. Notably, epidermal hyperplasia spontaneously resolved in global KO mice by 6-7 weeks of age (Figure 1E), whereas it persisted in cKO mice (Figure 1H). In contrast, barrier dysfunction, as measured by TEWL, remained elevated through 16 weeks of age in both mouse lines (Figure 1G and J). These data indicate that *Tmem79* expression in nonkeratinocytes appears to contribute to perturbed epidermal structure rather than its restoration.

### *Tmem79* loss induces hyperproliferation and impairs epithelial differentiation

To better understand epidermal defects observed in 4-week-old *Tmem79* cKO mice, we performed transmission electron microscopy (TEM). Consistent with histological findings, cKO skin showed a marked increase in epidermal thickness (SI Appendix, Figure S2C). Notably, the SC, the outermost layer that forms the primary skin barrier, was significantly expanded (Figure 2A). Although SC thickening does not necessarily indicate barrier failure, it has been associated with reduced barrier quality and increased TEWL in conditions such as cosmetic dry skin, ageing, and ichthyosis (26–29).

**Figure 2:**
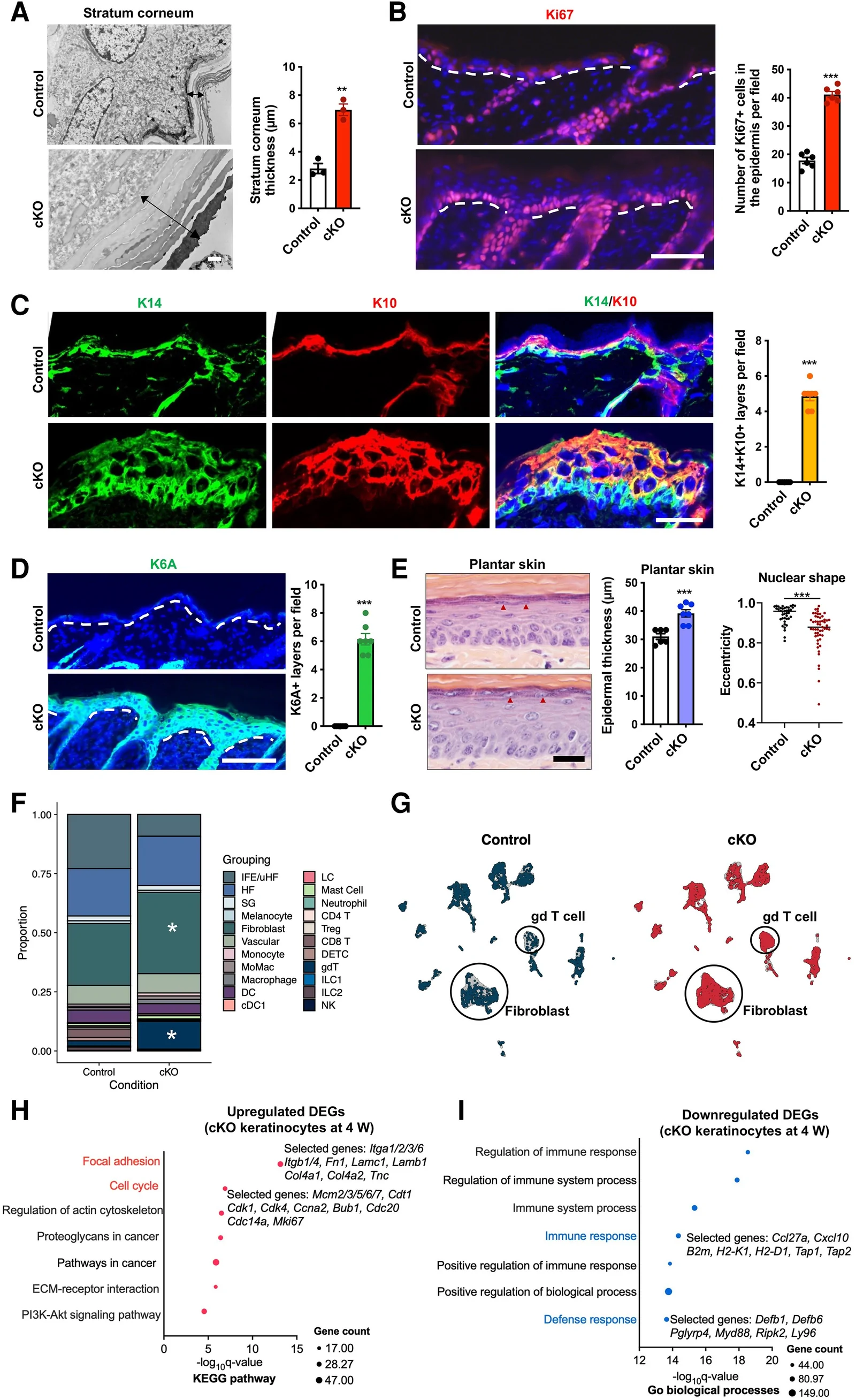
*Tmem79* deficiency drives keratinocyte hyperproliferation and impaired differentiation at 4 weeks of age. A) TEM images and quantification of stratum corneum thickness in control and cKO mice. Double-headed arrows indicate the thickness of the stratum corneum. Scale bar = 1 μm. n = 3 biological replicates for TEM. B) Immunostaining and quantification of the proliferation marker Ki67 in the epidermis of control and cKO mice. White dashed lines indicate epithelial–dermal borders. Scale bar = 50 μm. C) Immunostaining of K14 (green) and K10 (red), with quantification of K14^+^ and K10^+^ epidermal layers in control and cKO mice. Scale bar = 25 μm. D) Immunostaining and quantification of K6A in the epidermis of control and cKO mice. Scale bar = 50 μm. E) H&E images of plantar skin with quantification of epidermal thickness and nuclear shape eccentricity in the granular layer of control and cKO mice. Arrowheads indicate nuclei in the granular layer. Scale bar = 25 μm. F) Bar plots show the relative proportions of cell populations identified by scRNA-seq in whole skin from control and cKO mice (n = 2). Asterisks indicate elevated fibroblast and γδ T cell populations in cKO mice. G) UMAP plots of skin cells from control and cKO mice, with fibroblasts and γδ T cells highlighted. cKO skin exhibits expansion of both fibroblast and γδ T cell populations compared to control skin. H) KEGG pathway analysis of upregulated DEGs in cKO keratinocytes, with selected enriched pathways highlighted in red and associated genes indicated. I) GO biological process enrichment analysis of downregulated DEGs in cKO keratinocytes. Selected enriched terms are highlighted in blue, with corresponding gene sets shown.

Because the SC represents the terminal stage of epidermal differentiation and TMEM79 is mostly expressed in suprabasal keratinocytes, we next investigated whether differentiation was altered in cKO mice. Immunostaining for Ki67^+^ cells revealed a significant increase in proliferating cells within the cKO epidermis (Figure 2B). Whereas control skin showed proper spatial organization, with basal keratin 14 (K14) and suprabasal keratin 10 (K10) segregated into distinct layers, cKO mice showed co-expression of these markers, indicative of aberrant differentiation (Figure 2C). This pattern likely reflects abnormal transitional states between basal stem cells and differentiated keratinocytes (30, 31). In addition, the injury- and stress-associated marker K6A, which is normally absent from the interfollicular epidermis (IFE) (32, 33), was ectopically induced in the cKO epidermis (Figure 2D). These findings indicate that *Tmem79* deletion promotes stem cell proliferation and disrupted keratinocyte differentiation, likely contributing to the observed epidermal hyperplasia and barrier dysfunction.

By 4 weeks of age, global and cKO mice exhibited clear dorsal skin hair loss (SI Appendix, Figure S2D and E). Because hair follicle abnormalities and hair loss can secondarily influence epidermal architecture (34, 35), we sought to exclude the possibility that hair loss contributes to the observed epidermal hyperplasia. We therefore performed histological analyses of sparsely haired regions (ear and tail) as well as hairless plantar skin to determine whether hyperplasia occurs independently of hair follicles. H&E staining revealed a marked increase in epidermal thickness in all three regions (Figure 2E and SI Appendix, Figure S2F), ruling out a direct causative role for hair loss in driving epidermal hyperplasia.

Interestingly, within the granular layer, which is characterized by the presence of keratohyalin granules, nuclei in cKO mice remained round rather than elongated (Figure 2E). In normal epidermis, nuclear elongation, condensation, and enucleation accompanying terminal differentiation are required to establish the skin barrier (36, 37). The persistence of rounded nuclei in the granular layer of cKO mice suggests impaired nuclear remodeling and defective terminal differentiation.

At 4 weeks of age, keratinocyte-specific *Tmem79* deletion also caused histological features of dermal immune cell infiltration (Figure 1I). To better understand the cellular changes in the skin microenvironment, we performed scRNA-seq of dorsal skin from 4-week-old control and cKO mice (SI Appendix, Figure S3A). This identified 22 distinct cell populations, with *Tmem79* cKO skin showing increased proportions of γδ T cells and fibroblasts (SI Appendix, Figure S3B and Figure 2F–G), consistent with immune activation and remodeling of the skin microenvironment. To understand keratinocyte-intrinsic transcriptional changes, we performed Kyoto Encyclopedia of Genes and Genomes (KEGG) pathway and Gene Ontology (GO) analysis on keratinocyte-specific differentially expressed genes (DEGs) from the scRNA-seq dataset between *Tmem79* cKO and control mice. KEGG analysis revealed enrichment of focal adhesion and cell cycle pathways, with the cell cycle pathway supported by increased expression of *Mcm2, Mcm3, Mcm5, Mcm6, Mcm7, Cdt1, Cdk1, Cdk4, Ccna2, Bub1, Cdc20, Cdc14a, and Mki67* (Figure 2H). GO analysis showed enrichment of pathways related to actin cytoskeleton organization, epithelial morphogenesis, and cell junction organization (SI Appendix, Figure S3C), consistent with impaired epithelial differentiation and barrier dysfunction. In contrast, genes involved in immune and defense responses were downregulated in cKO keratinocytes, including *Ccl27a, Cxcl10, B2m, H2-K1, H2-D1, Tap1, Tap2, Defb1, Defb6, Pglyrp4, Myd88, Ripk2, and Ly96* (Figure 2I). Consistently, KEGG analysis showed reduced enrichment of pathways related to antigen processing and presentation and virus infection–related pathways (SI Appendix, Figure S3D).

Together, these data indicate that *Tmem79* deficiency reprograms keratinocytes toward a hyperproliferative and structurally remodeled state while compromising epithelial immune defense programs.

### TMEM79 loss impairs human keratinocyte differentiation and barrier formation

To assess whether the keratinocyte-intrinsic consequences of TMEM79 loss are conserved in a human system, we used N/TERT-1 human keratinocytes, which are capable of full epidermal differentiation and are amenable to genetic editing. We generated three independent TMEM79 knockout and control clones using CRISPR/Cas9. Biallelic disruption was confirmed using Sanger sequencing (SI Appendix, Figure S4) and TMEM79 protein loss by western blot analysis (SI Appendix, Figure S5A).

To define transcriptional programs associated with TMEM79 deficiency and characterize the knockout lines, we performed bulk RNA-seq under two conditions: a proliferative basal-like state (nonconfluent, 0.3 mM CaCl_2_) and a differentiative state (3 days at confluence, 1.2 mM CaCl_2_). Upon differentiation, TMEM79-deficient cells downregulated genes associated with keratinocyte differentiation and keratinization, including *ZNF750*, *SPRR1A*, *SPRR2D*, *SPRR2E*, *LCE3D*, *LCE3E*, *CDSN*, *CNFN*, *ALOXE3*, *ALOX12B* (SI Appendix, Figure S5B–D), indicating intrinsic deficits in differentiation.

We next asked whether impaired differentiation translated into reduced barrier formation. Control and TMEM79-null clones were differentiated in air–liquid interface (ALI) culture to form 3D human epidermal equivalents (HEEs) (38). Transepithelial electrical resistance (TEER) was measured as a quantitative, noninvasive readout of barrier integrity (38, 39). After 10 days of ALI culture, TMEM79-deficient HEEs exhibited a 53% reduction in TEER relative to controls (SI Appendix, Figure S5E). However, EdU incorporation assays showed no differences in proliferation between TMEM79-null and control N/TERT-1 cells (SI Appendix, Figure S5F). These data indicate that TMEM79 deficiency impairs human keratinocyte differentiation and barrier formation without increasing proliferation.

We previously showed that TMEM79 antagonizes Wnt signaling by promoting Frizzled degradation (23). Consistent with this function, TMEM79 loss in human keratinocytes significantly increased cytosolic active β-catenin levels (SI Appendix, Figure S6A) and expression of the Wnt target gene *AXIN2* (SI Appendix, Figure S6B), which we observed across independent clones. These findings suggest that Wnt/β-catenin dysregulation may contribute to the differentiation defects associated with TMEM79 deficiency.

### TMEM79 deficiency causes decreased viability and ferroptotic stress in keratinocytes

Although TMEM79-null N/TERT-1 clones displayed similar proliferative capacity (SI Appendix, Figure S5F), they consistently yielded fewer cells upon passaging, suggesting a defect in cell survival. Transcriptional analysis of control and TMEM79 KO keratinocytes under basal-like conditions showed upregulation of genes related to cell death pathways (Figure 3A and B), including *RBCK1*, *PNMA1, PDCD4, PHLDA1, EGR1, TXNIP, FOS,* and *PTGS2* (Figure 3C). Consistent with this, the cell counting kit-8 (CCK8) assay and clonogenic potential assays confirmed reduced viability and colony-forming potential in TMEM79-deficient clones (Figure 3D and E). Increased keratinocyte cell death was also observed in the epidermis of *Tmem79* cKO mice by TEM and terminal deoxynucleotidyl transferase dUTP nick end labeling (TUNEL) staining (SI Appendix, Figure S7A and B). To quantify cell death in the human keratinocytes, we measured lactate dehydrogenase (LDH) release as a readout of membrane integrity and found that TMEM79 KO cells released significantly higher LDH levels than controls (Figure 3F).

**Figure 3:**
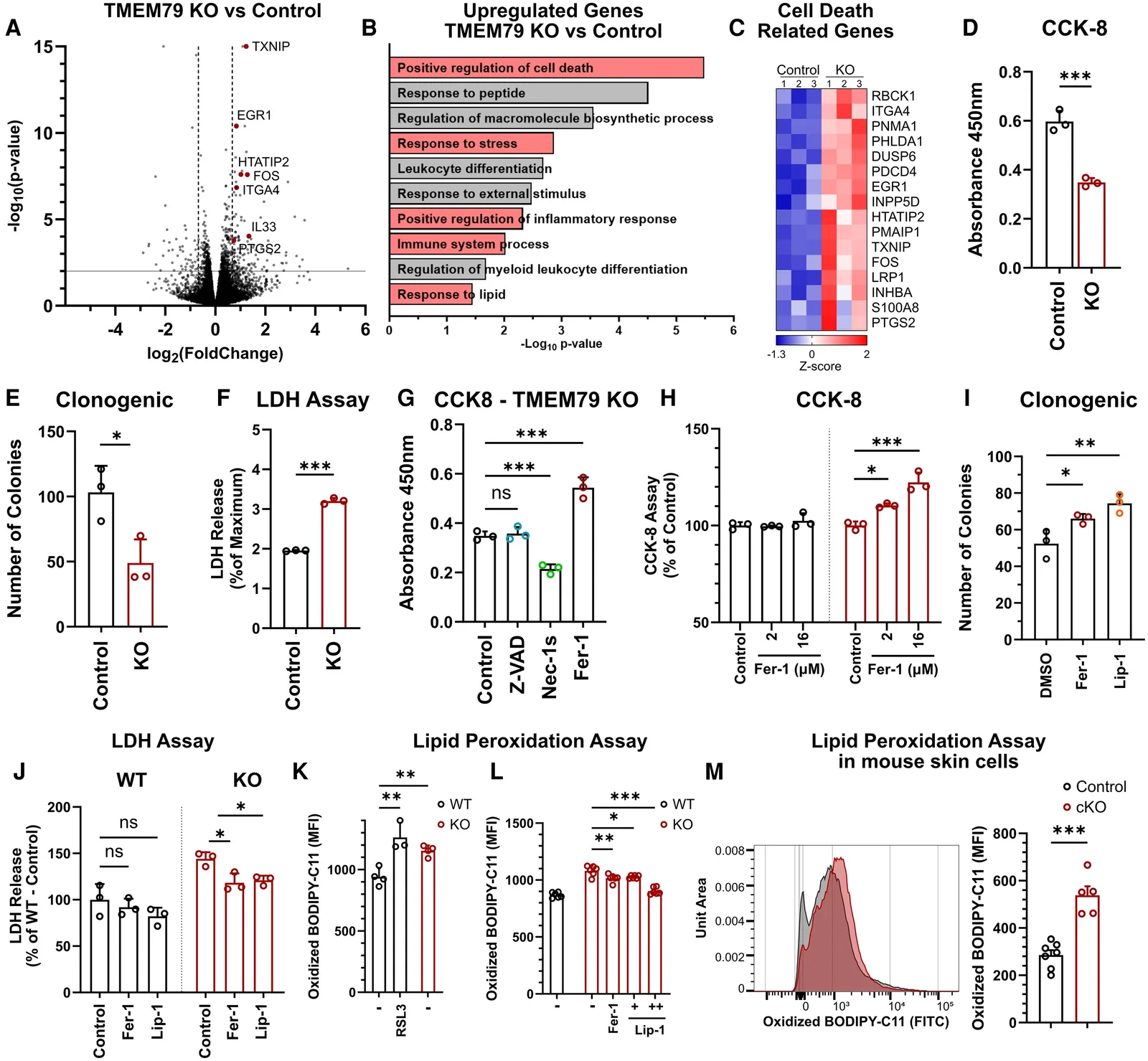
TMEM79 deficiency induces ferroptotic cell death and lipid peroxidation in keratinocytes. A) Volcano plot of differentially expressed genes in control and TMEM79-deficient N/TERT-1 cells under undifferentiated conditions. Upregulated genes associated with inflammatory and cellular stress responses are highlighted in red. B) Gene Ontology (GO) enrichment analysis of genes upregulated in TMEM79-deficient cells, revealing enrichment of pathways related to cellular stress and regulation of cell death. C) Heatmap showing increased expression of selected cell death–associated genes in TMEM79-deficient cells compared to controls. D) Cell viability assessed by CCK8 assay in control and TMEM79-deficient N/TERT-1 cells. E) Clonogenic assay demonstrating reduced colony-forming capacity of TMEM79-deficient cells relative to controls. F) Lactate dehydrogenase (LDH) release assay showing increased cytotoxicity in TMEM79-deficient cells compared to controls. G) CCK8 viability assay of TMEM79-deficient cells treated with vehicle (DMSO), Z-VAD-FMK (apoptosis inhibitor), Necrostatin-1s (Nec-1s; necroptosis inhibitor), or Ferrostatin-1 (Fer-1; ferroptosis inhibitor). H) CCK8 assay showing dose-dependent rescue of viability in TMEM79-deficient cells treated with Fer-1 (2 or 16 μM), with wild-type cells shown in black and knockout cells in red. I) Clonogenic assay of TMEM79-deficient cells treated with Fer-1 (2 μM) or Liproxstatin-1 (Lip-1; 0.2 μM), demonstrating improved clonogenic survival upon ferroptosis inhibition. J) LDH release assay of wild-type and TMEM79-deficient cells treated with Fer-1 or Lip-1, showing reduced cytotoxicity in knockout cells. K) BODIPY-C11 lipid peroxidation assay showing increased oxidized BODIPY-C11 signal in TMEM79-deficient cells compared to wild-type controls. RSL3 was used as a positive lipid peroxidation control. L) BODIPY-C11 assay demonstrating reduced lipid peroxidation in TMEM79-deficient cells following treatment with Fer-1 (2 μM) or Lip-1 (0.2 or 2 μM). M) BODIPY-C11 lipid peroxidation analysis of epidermal sheet cells isolated from control and *Tmem79* cKO mice. Concatenated histograms depict the median fluorescence intensity of the FITC channel. Data are shown as mean ± SEM; ***P < 0.001 (unpaired t-test); n = 7 control mice and n = 5 cKO mice. Statistical significance between two groups was determined using an unpaired t-test. Comparisons among multiple groups were analyzed by ordinary one-way ANOVA followed by Dunnett's multiple comparisons test. Data are presented as mean ± SD unless otherwise indicated. *P < 0.05, **P < 0.01, ***P < 0.001, ns, not significant.

To define which cell death pathway contributes to this phenotype, we treated TMEM79 KO cells with pharmacological inhibitors that block apoptosis (Z-VAD-FMK), necroptosis (Necrostatin-1s), or ferroptosis (Ferrostatin-1 or Fer-1). Only Fer-1 significantly improved viability in TMEM79-null cells, implicating ferroptosis in keratinocyte death (Figure 3G). We next asked whether ferroptosis inhibition restores functional viability. We evaluated the ferroptosis inhibitors Fer-1 and Liproxstatin-1 (Lip-1) in viability and clonogenic assays. Fer-1 rescued cell viability in TMEM79-deficient keratinocytes in a dose-dependent manner (Figure 3H). Both inhibitors enhanced colony-forming capacity and reduced LDH release from TMEM79-null cells to near-control levels (Figure 3I and J). These data identify ferroptosis as a major contributor to cell death caused by TMEM79 deficiency. Because ferroptosis is driven by iron-dependent lipid peroxidation (40), we next measured lipid oxidation using the BODIPY-C11 probe, which shifts fluorescence from red to green upon oxidation (41). TMEM79-deficient keratinocytes exhibited markedly increased oxidized BODIPY-C11 fluorescence relative to controls, reaching levels comparable to direct induction of ferroptosis in control cells with the GPX4 inhibitor RSL3 (Figure 3K). Treatment with Fer-1 or Lip-1 significantly reduced oxidized BODIPY-C11 levels in TMEM79-null cells, supporting ferroptosis-associated lipid peroxidation as a driver of cell death caused by TMEM79 deficiency (Figure 3L).

To determine whether lipid peroxidation was also present in vivo, we evaluated epidermal sheets from 4-week-old *Tmem79* cKO and control mice using oxidized BODIPY-C11. *Tmem79* cKO epidermis displayed increased lipid peroxidation relative to controls (Figure 3M). Consistent with this observation, keratinocytes from *Tmem79* cKO mice showed increased expression of genes associated with ferroptosis and lipid metabolism, including *Fbxl5, Ftl1, Slc39a14, Pla2g7, Acsl3, Lpin2,* and *Lclat1* (SI Appendix, Figure S7C). Therefore, ferroptotic stress is a major component of the keratinocyte response to TMEM79 loss in both human cells and the mouse model.

### Type 17 immunity drives epidermal hyperplasia

Having established that TMEM79 loss impairs keratinocyte differentiation and barrier formation, we next asked whether epithelial dysfunction leads to immune responses that amplify skin pathology. Whole-skin scRNA-seq analysis showed a marked increase in γδ T cells at 4 weeks of age, the peak of disease in *Tmem79* cKO mice (Figure 2F and G). To define this immune response in greater depth, we sorted CD45^+^ immune cells and performed scRNA-seq to profile immune populations that are underrepresented in whole-skin datasets. Single-cell analysis identified 16 distinct CD45^+^ immune populations, with cKO mice showing increased proportions of γδ T cells, neutrophils, and mast cells (SI Appendix, Figure S8A and B and Figure 4A). Neutrophil infiltration was confirmed by flow cytometry (SI Appendix, Figure S8C). Immunostaining for the neutrophil marker myeloperoxidase (MPO) showed that neutrophils were predominantly located in the lower dermis in control skin but were positioned closer to the epidermis in cKO skin (SI Appendix, Figure S8D). This positioning is consistent with increased expression of neutrophil-recruiting chemokines by cKO keratinocytes, such as CXCL1, as detected by both scRNA-seq and qPCR (SI Appendix, Figure S8E and F). Mast cell infiltration in cKO mice was confirmed by toluidine blue staining (SI Appendix, Figure S8G), consistent with a previous report that mast cells are increased and mediate histaminergic itch in *Tmem79* KO mice (20).

**Figure 4:**
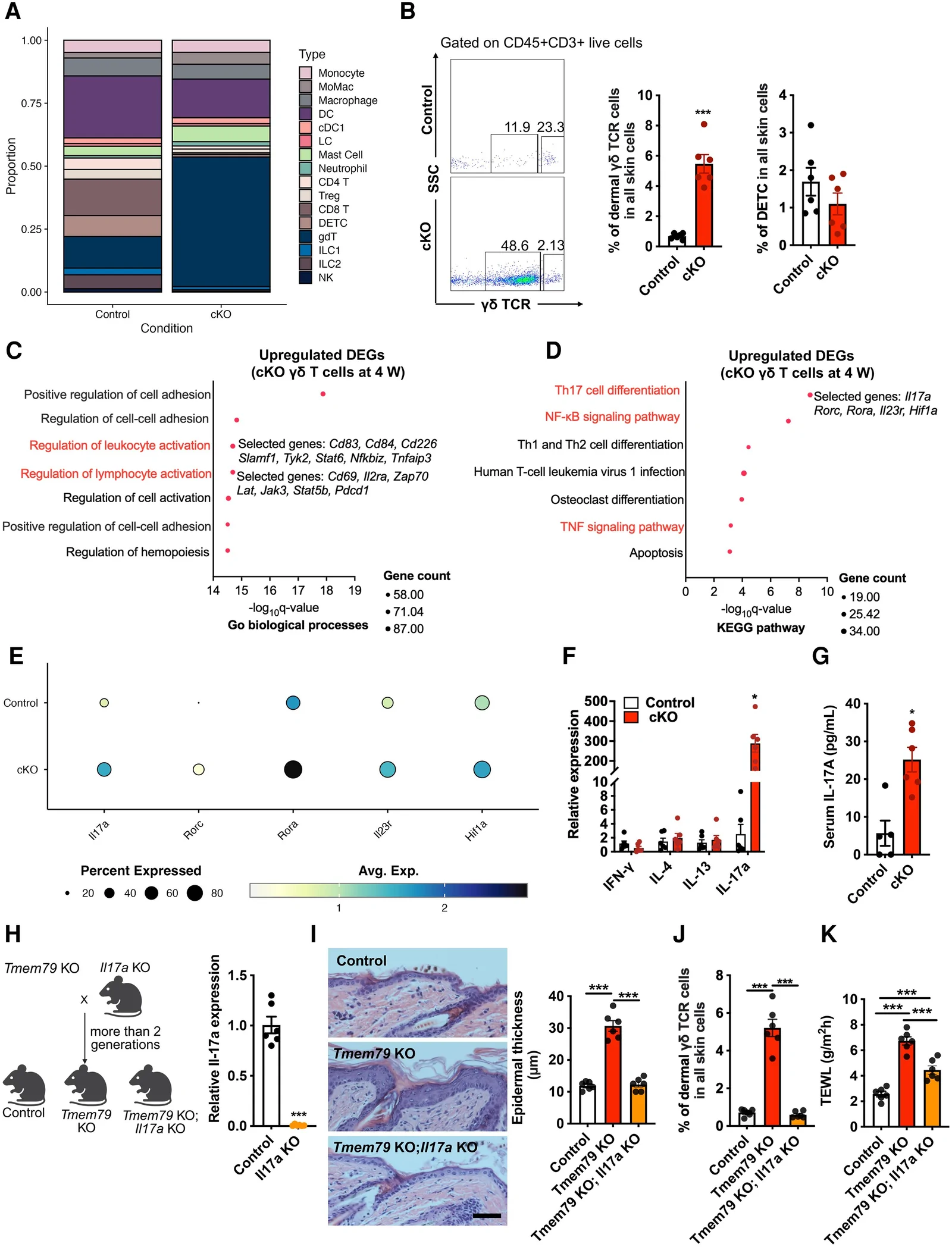
Type 17 immunity drives epidermal hyperplasia. A) Bar plots show the relative proportions of immune cell populations identified by scRNA-seq in CD45^+^ FACS-sorted cells from 4-week-old control and cKO mice (n = 2). B) FACS profiling and quantification of γδ TCR in skin from 4-week-old control and cKO mice. Dermal γδ T cells are characterized by low to intermediate γδ TCR expression, whereas dendritic epidermal T cells (DETCs) exhibit high γδ TCR expression. C) GO biological process analysis of upregulated DEGs in cKO γδ T cells, with selected enriched terms highlighted in red and their associated gene sets. D) KEGG pathway analysis of upregulated DEGs in cKO γδ T cells, with selected enriched pathways highlighted in red and corresponding gene sets shown. E) Dot plot showing the expression of Th17-associated genes (*Il17a, Il23r, Rorc, Rora, and Hif1a*) in γδ T cells from 4-week-old control and cKO mice. F) qPCR analysis of *IFN-γ*, *IL-4*, *IL-13*, and *IL-17a* expression in 4-week-old control and cKO skin. G) ELISA analysis of serum IL-17A levels in 4-week-old control and cKO mice. H) Schematic for generating *Tmem79* and *Il17a* double KO mice, and qPCR analysis of *Il17a* expression in 4-week-old control and *Il17a* KO mice. The schematic was created using BioRender. I–K) H&E images and quantification of epidermal thickness (I), dermal γδ TCR^+^ cells (J), and TEWL (K) in 4-week-old control (*Tmem79+/−; Il17a+/−*), *Tmem79* KO, and *Tmem79/Il17a* double KO mice. Scale bar = 50 μm. For comparisons among more than two groups in all mouse experiments, data are presented as mean ± SEM. Statistical significance was determined by ordinary one-way ANOVA followed by Tukey's multiple comparisons test; *P < 0.05, **P < 0.01, ***P < 0.001.

Flow cytometry confirmed the increase in dermal γδ T cells in cKO skin (Figure 4B). We next performed pseudobulk differential expression analysis to define their transcriptional state. cKO γδ T cells displayed a strongly activated and inflammatory profile. GO analysis of DEGs revealed enrichment of pathways related to leukocyte and lymphocyte activation, supported by increased expression of *Cd83, Cd84, Cd226, Slamf1, Tyk2, Stat6, Nfkbiz, Tnfaip3, Cd69, Il2ra, Zap70, Lat, Jak3, Stat5b, and Pdcd1* (Figure 4C). KEGG analysis further indicated signs of γδ-Th17 cell differentiation, with upregulation of *Il17a, Rorc, Rora, Il23r, and Hif1a* (Figure 4D and E), along with enrichment of NF-κB and TNF signaling pathways (Figure 4D). In contrast, downregulated DEGs point to a suppression of cytoplasmic translation, ribosome function, and natural killer cell–mediated cytotoxicity, suggesting a shift away from a homeostatic and cytotoxic program (SI Appendix, Figure S8H and I). Together, these findings indicate that loss of *Tmem79* in keratinocytes drives γδ T cells toward an activated, IL-17–skewed effector state.

The expansion of γδ T cells in cKO skin is likely not a result of local proliferation, as immunofluorescence showed distinct γδ TCR and Ki67 signals (SI Appendix, Figure S8J). This suggests a potential recruitment of γδ T cells, likely from the lymph nodes, consistent with increased frequencies of γδ T cells in skin-draining lymph nodes of cKO mice (SI Appendix, Figure S8K). Analysis of skin cytokine levels revealed significant elevation of *Il-17a*, unchanged type 2 cytokines (*Il-4*, *Il-13*), and a nonsignificant downward trend in *IFN-γ* (*P* = 0.15) (Figure 4F). Serum IL-17A was also increased (Figure 4G). These findings indicate that 4-week-old cKO mice show a type 17-dominant immune response, consistent with the γδ T cell transcriptional signatures (Figure 4C–E).

To test whether suppressing type 17 immunity could ameliorate TMEM79-associated phenotypes, we generated *Tmem79* KO; *Il17a* KO mice (Figure 4H). These double KO mice exhibited a marked reduction in epidermal hyperplasia and dermal γδ T cell infiltration to levels comparable to control mice (Figure 4I and J). Although skin barrier dysfunction was significantly improved, it was not fully restored to baseline (Figure 4K). These findings, together with data from TMEM79 KO human keratinocytes (SI Appendix, Figure S5), indicate that early skin barrier dysfunction is largely driven by keratinocyte-intrinsic factors, whereas epidermal hyperplasia is primarily driven by IL-17A dependent immune responses that further exacerbate barrier dysfunction.

### Early IFN signaling and keratinocyte changes precede type 17 inflammation

Because IL-17A contributed mainly to peak disease at 4 weeks of age, we next asked whether key earlier epithelial and immune-related programs emerge before overt skin pathology. To define these initiating events, we examined epithelial abnormalities in the epidermis at time points prior to the scaling, hyperplasia and skin barrier dysfunction observed at 2 weeks of age (Figure 5A–D). We performed longitudinal bulk RNA-seq profiling of epidermal sheets isolated from control and cKO mice at P1 (postnatal day 1), P3, P6, and P14 (Figure 5E). Aside from *Tmem79*, no DEGs were observed at P1, indicating that cutaneous pathology develops postnatally rather than embryonically. The number of DEGs then increased from 13 at P3 to 136 at P6 and to 113 at P14 (Figure 5E).

**Figure 5:**
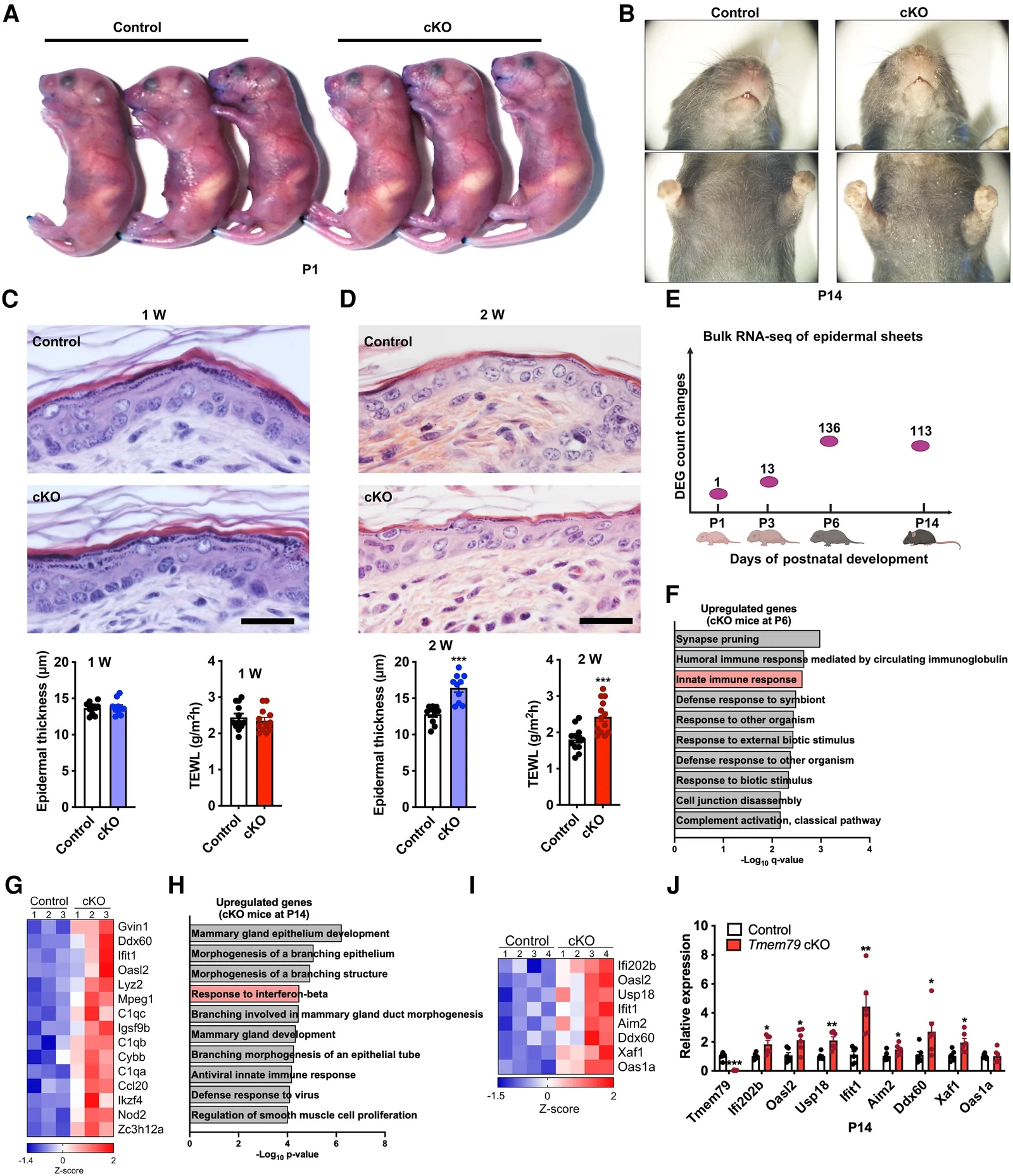
Increased type I IFN signaling during early life in *Tmem79* cKO mice. A) Toluidine blue dye penetration assay at P1 indicates intact epidermal barrier formation in cKO mice. B) Photographs of control and cKO mice at P14 showing prominent skin scaling in cKO mice. C) H&E images, quantification of epidermal thickness, and TEWL in control and cKO mice at 1 W. Scale bar = 25 μm. D) H&E images, quantification of epidermal thickness, and TEWL in control and cKO mice at 2 W. Scale bar = 25 μm. E) Bulk RNA-seq of epidermal sheets collected at P1-P14 shows the number of DEGs. Numbers above each point denote total DEG counts. F) GO analysis of upregulated DEGs in cKO epidermis at P6, with selected enriched term highlighted in red. G) Heatmaps depicting the increased expression of some genes associated with the innate immune response in cKO epidermis at P6. H) GO analysis of upregulated DEGs in cKO epidermis at P14, with selected enriched term highlighted in red. I) Heatmaps illustrating the heightened expression of IFN-related genes in cKO epidermis at P14. J) qPCR analysis of *Tmem79* and type I IFN-related genes identified by bulk RNA-seq in control and cKO epidermis at P14. Except for *Oas1a*, all other genes show significant changes in cKO epidermis.

At P3, beyond *Tmem79*, the other 12 DEGs remain poorly characterized in the context of skin biology or pathology (SI Appendix, Figure S9A). At P6 and P14, downregulated genes were significantly enriched for processes related to epidermal development and keratinization (SI Appendix, Figure S9B and C), consistent with the differentiation and barrier defects observed in TMEM79-deficient human keratinocytes and adult cKO skin. In parallel, genes upregulated at P6 were enriched for innate immune response, including early induction of IFN-stimulated genes such as *Gvin1*, *Ddx60*, *Ifit1*, *Oasl2*, suggesting the initial activation of type I IFN signaling (Figure 5F and G). This pattern became more pronounced at P14, with the emergence of a robust type I IFN response signature characterized by genes such as *Ifi202b*, *Oasl2*, *Usp18*, *Ifit1*, *Aim2*, *Ddx60*, and *Xaf1*, which we further validated by qPCR (Figure 5H–J). Together, this temporal profiling indicates that *Tmem79* loss induces early epidermal differentiation defects together with a type I IFN response, preceding the later type 17 inflammatory response.

To define the cellular basis of these early changes, we performed scRNA-seq on dissociated skin collected at P6, an early time point corresponding to the emergence of numerous DEGs in cKO epidermal sheets (Figure 5E). We observed an increased proportion of proliferating interfollicular epidermal (IFE) cells in cKO skin (SI Appendix, Figure S10A and B). Differential expression analysis across keratinocytes from cKO and control skin revealed a strong induction of cell cycle and mitosis-related genes like *Top2a*, *Mki67*, and *Wee1,* consistent with heightened proliferative activity (SI Appendix, Figure S10C). Inflammatory and stress–response genes such as *Tnfaip3* and *Nod2* were also upregulated, reflecting an inflammatory-activated state (SI Appendix, Figure S10C). In contrast, genes involved in epidermal differentiation *(Krt10*, *Krt77, Ivl*) and regulators of epidermal homeostasis and barrier integrity (*Zfp36*, *Igfbp3*) were significantly downregulated in P6 keratinocytes (SI Appendix, Figure S10C). The increased proliferative activity and impaired epidermal differentiation in cKO IFE are consistent with our earlier observations during peak disease (Figure 2).

### Langerhans cells are dispensable for TMEM79-associated skin pathology

Beyond keratinocytes, P6 cKO skin showed a nonsignificant trend toward increased Langerhans cells (LCs) and dendritic epidermal T cells (DETCs), which becomes more pronounced at peak disease at 4 weeks of age (SI Appendix, Figure S10B and S10D). We found that the inflammatory genes upregulated in P6 cKO epidermal sheets (Figure 5G) were predominantly expressed by LCs and not DETCs nor keratinocytes (SI Appendix, Figure S10E). These genes (*Lyz2*, *C1qc*, *C1qb*, *Cybb*, and *C1qa*) are associated with phagocytic activity, indicating potentially enhanced LC activation (42–44). LCs can migrate to draining lymph nodes, where they present antigens and promote immune activation, including type 17 responses (45, 46). DETCs showed elevated expression of *Tnf*, *Egr1*, *Il13* and *Ifng* in P6 cKO skin (SI Appendix, Figure S10F). However, previous work showed that DETC-deficient *Tcrd* KO mice did not impact initial-phase dermatitis (<7 weeks) (19).

We therefore investigated whether LC activation may serve as an upstream trigger of skin pathology or the immune response in *Tmem79* cKO mice. To test this, we crossed *Tmem79* cKO mice with Langerin-diphtheria toxin A (DTA) mice to ablate LCs (SI Appendix, Figure S10G and H). LC depletion did not affect epidermal hyperplasia, barrier dysfunction, or the type 17 immune response in cKO mice (SI Appendix, Figure S10I–K). These results indicate that while we see changes in LCs, their activation is not a direct driver of the pathological features of *Tmem79* cKO mice. Instead, they support a model in which immune activation arises as a consequence of keratinocyte-intrinsic changes and epithelial stress.

### STING-dependent signaling promotes type 17 inflammation and skin pathology

The progressive emergence of a type I IFN gene signature in the epidermis from P6 to P14 (Figure 5F–J) suggested that this pathway may contribute to the later development of TMEM79-associated skin pathologies. To test this, we crossed *Tmem79* cKO mice with *Ifnar1* KO mice, which lack functional type I IFN receptors (Figure 6A). *Tmem79* cKO; *Ifnar1* KO mice exhibited reduced epidermal hyperplasia, improved barrier integrity, and a diminished type 17 immune response, as evidenced by decreased γδ T cell infiltration and lower *Il17a* expression (Figure 6B and C and SI Appendix, Figure S11A and B). These findings support a role for early type I IFN signaling in driving TMEM79-associated skin and immune pathologies.

**Figure 6:**
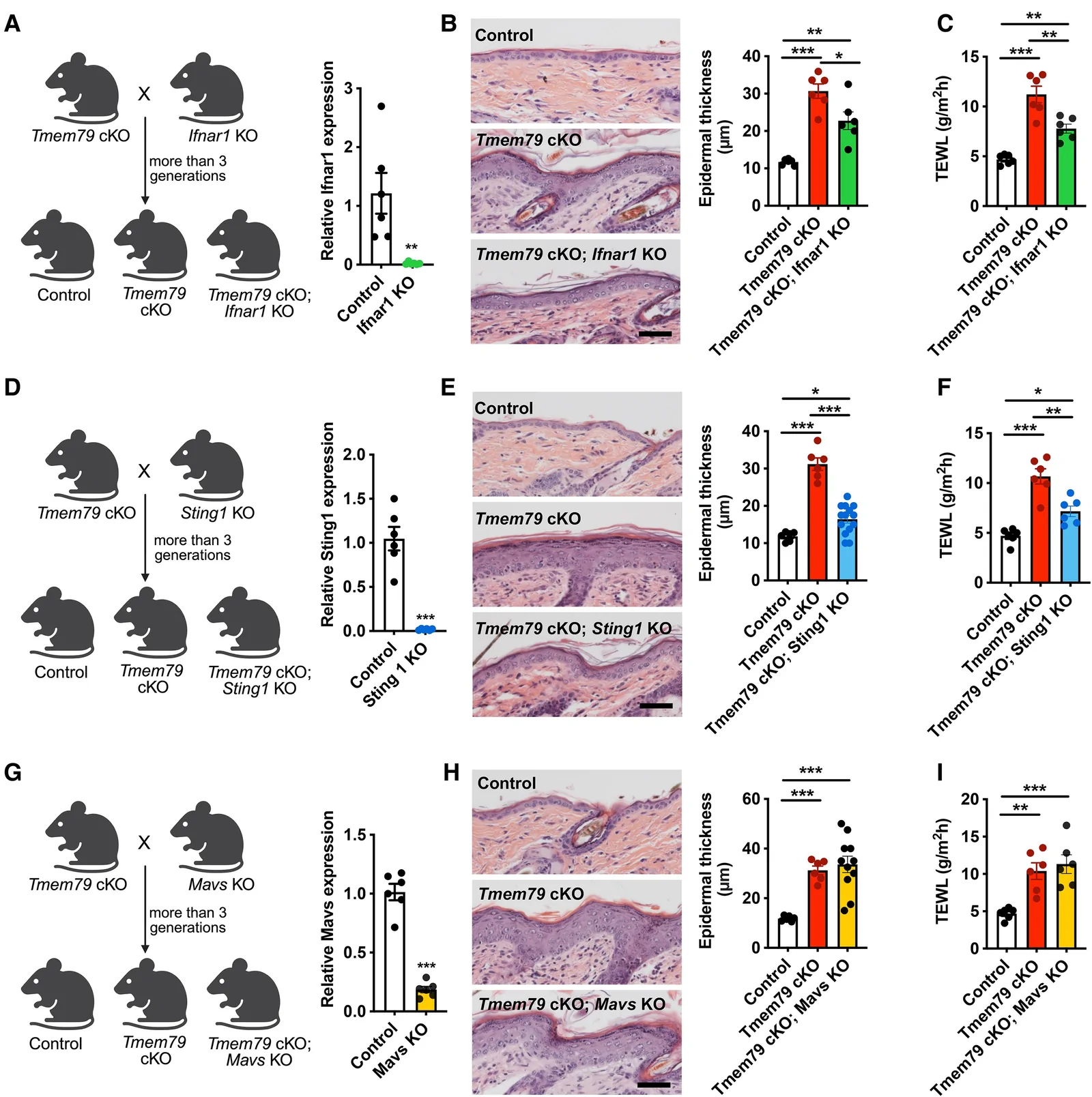
Early STING-dependent signaling drives the cutaneous pathological features in *Tmem79* cKO mice. A) Schematic illustrating the generation of *Tmem79* cKO*; Ifnar1* KO mice, with qPCR analysis of *Ifnar1* expression in 4-week-old control and *Ifnar1* KO mice. B–C) Representative H&E-stained sections and quantification of epidermal thickness (B) and TEWL (C) are shown for 4-week-old control (*Tmem79 fl/+; Ifnar1+/−* or *Tmem79 fl/fl; Ifnar1+/−*), *Tmem79* cKO, and *Tmem79* cKO; *Ifnar1* KO mice. D) Schematic of the generation of *Tmem79* cKO; *Sting1* KO mice, with qPCR validation of *Sting1* deletion at 4 weeks of age. E–F) H&E-stained images and quantification of epidermal thickness (E) and TEWL (F) in 4-week-old control (*Tmem79 fl/+* or *Tmem79 fl/fl*), *Tmem79* cKO, and *Tmem79* cKO; *Sting1* KO mice. G) Schematic for generating *Tmem79* cKO; *Mavs* KO mice, with qPCR validation of *Mavs* deletion at 4 weeks of age. H–I) H&E images and quantification of epidermal thickness (H) and TEWL (I) in 4-week-old control (*Tmem79 fl/+; Mavs+/−* or *Tmem79 fl/fl; Mavs+/−*), *Tmem79* cKO, and *Tmem79* cKO; *Mavs* KO mice. Scale bars, 50 µm (B, E, H). All schematics in the figure were created using BioRender.

Type I IFNs can be induced downstream of multiple nucleic acid sensors, including the cGAS/STING pathway, which detects intracellular DNA, and the RIG-I/MAVS pathway, which senses double-stranded RNA (47–49). To identify the key molecular sensors upstream of type I IFN induction in *Tmem79* cKO skin, we crossed *Tmem79* cKO mice with *Sting1* KO or *Mavs* KO mice. *Tmem79* cKO; *Sting1* KO mice exhibited reduced epidermal hyperplasia, improved barrier function, and diminished type 17 responses (Figure 6D–F and SI Appendix, Figure S11C and D). By contrast, loss of *Mavs* did not alter these phenotypes in *Tmem79* cKO mice (Figure 6G–I and SI Appendix, Figure S11E).

Together, these findings indicate that a STING-mediated, MAVS-independent mechanism drives a type I IFN response during early life and underlies the development of persistent epidermal hyperplasia, barrier dysfunction, and type 17 immune activation observed in adult *Tmem79* cKO mice.

Overall, these findings indicate that *Tmem79* cKO mice spontaneously develop a STING-dependent response that promotes a subsequent type 17 immune response. TMEM79 deficiency in human keratinocytes captures several epithelial-intrinsic features of *Tmem79* cKO skin, including impaired differentiation, defective barrier formation, reduced cell viability, and ferroptotic lipid peroxidation. However, TMEM79-deficient human keratinocytes did not exhibit increased proliferation, and we did not detect a type I IFN signature or STING activation in vitro. Thus, while TMEM79 loss is sufficient to induce epithelial stress responses in human keratinocytes, epidermal hyperplasia and inflammatory activation in vivo likely require additional tissue-level cues.

## Discussion

Our study identifies TMEM79 as a keratinocyte-intrinsic regulator that integrates epidermal differentiation, barrier formation, and epithelial stress responses. By combining keratinocyte-specific genetic ablation in mice with human keratinocyte models, we show that loss of TMEM79 disrupts epidermal differentiation and barrier formation, induces ferroptotic lipid peroxidation, and is associated in vivo with early STING-dependent type I interferon signaling. This epithelial-intrinsic stress program precedes the emergence of barrier dysfunction and a type 17 immune response, which in turn drives epidermal hyperplasia (SI Appendix, Figure S12). Together, our findings indicate that keratinocyte-intrinsic defects in barrier formation and lipid stress are associated with innate immune activation and progression of inflammatory skin disease.

Previous studies have demonstrated that mutations or global deletion of *Tmem79* lead to spontaneous dermatitis (16, 17, 19). Work from the Julius laboratory dissected tissue-specific contributions of TMEM79, showing that keratinocyte-specific loss was sufficient to induce skin inflammation, as evidenced by increased CD45^+^ immune cell infiltration and mast cell accumulation in the dermis (20). Work from the Schwartz group further demonstrated that K14-Cre*; Tmem79^fl/fl^* mice develop both spontaneous skin and lung inflammation (21). Consistent with these findings, our keratinocyte-specific deletion experiments show that epidermal loss of TMEM79 is sufficient to recapitulate major pathological features observed in global knockout mice, including epidermal hyperplasia, barrier dysfunction, and type 17 immune activation. Notably, epidermal hyperplasia persists in keratinocyte-specific knockout mice even as it partially resolves in global knockouts, suggesting that *Tmem79* in nonepithelial compartments contributes to disease progression. Collectively, these studies support an epithelial origin for TMEM79-associated pathology, but the keratinocyte-intrinsic mechanisms that initiate epithelial stress and license downstream immune activation remained unresolved.

Our study addresses this gap by defining epithelial differentiation defects, Wnt/β-catenin dysregulation, ferroptotic lipid stress, and STING-dependent type I IFN signaling as mechanisms linking TMEM79 deficiency to inflammatory skin pathology. *In vivo*, *Tmem79* cKO keratinocytes displayed hyperproliferation, aberrant K14/K10 organization and impaired terminal differentiation. However, TMEM79-deficient human keratinocytes exhibited no differences in proliferation, indicating that hyperproliferation is not a cell-intrinsic consequence of TMEM79 loss. Instead, our data suggest that TMEM79 plays a primary role in keratinocyte differentiation and barrier formation, whereas epidermal hyperplasia is driven by the inflammatory tissue environment. This interpretation is supported by the marked reduction in epidermal thickness in *Tmem79* KO; *Il17a* KO mice, consistent with prior studies showing that IL-17 acts on keratinocytes to induce proliferative and inflammatory programs in psoriasis (50). The barrier defect is evident both in vivo, where *Tmem79* cKO mice showed increased TEWL, and in vitro, where TMEM79-deficient HEEs show reduced TEER. *TMEM79* expression also increased during keratinocyte differentiation, supporting a role in late-stage epidermal maturation rather than basal stem cell maintenance.

Temporal transcriptomic profiling of *Tmem79* cKO mice revealed increased type I IFN signaling in the epidermis at early postnatal ages. Genetic ablation of *Ifnar1* in *Tmem79* cKO mice substantially rescued epidermal hyperplasia, improved barrier function, and reduced type 17 immune responses. Type I IFN signaling has been extensively implicated in human inflammatory skin diseases, including psoriasis and cutaneous lupus, where IFN-inducible gene signatures are strongly upregulated in lesional skin and contribute to pathogenic immune activation (51–55). Together, these findings position type I IFN signaling as a critical molecular bridge linking epithelial stress to the development of chronic inflammation.

Mechanistically, we identify STING as the dominant innate sensor driving skin inflammation in *Tmem79* cKO mice. Although induction of type I IFN responses is a major outcome of STING activation, STING can also promote proinflammatory cytokine production through type I IFN–independent mechanisms, including NF-κB signaling (48, 56). The diminished type 17 immunity in *Tmem79* cKO; *Sting1* KO mice is more pronounced, nearly comparable to control mice, than in *Tmem79* cKO; *Ifnar1* KO mice, suggesting that STING functions through both type I IFN–dependent and type I IFN–independent proinflammatory mechanisms. Genetic deletion of *Sting1*, but not the RNA-sensing adaptor *Mavs*, attenuated epidermal pathology and immune activation, indicating that cytosolic DNA sensing rather than MAVS-dependent RNA sensing is the dominant innate sensing pathway in this model. Although the precise source of immunostimulatory DNA remains to be defined, our data establish a STING-mediated, MAVS-independent pathway as the central driver of early IFN responses. Consistent with this framework, the cGAS–STING pathway has emerged as a critical mediator of cutaneous inflammation in experimental models of lupus and psoriasis (57–59).

How TMEM79 loss engages STING activation remains unclear. One consequence of TMEM79 deficiency we found in vitro is ferroptotic lipid stress. Ferroptosis is a regulated form of cell death driven by iron-dependent lipid peroxidation (40, 60), and has recently been implicated in inflammatory diseases (61, 62). In our study, human TMEM79-null keratinocytes exhibit increased lipid peroxidation and reduced viability, both reversed by ferroptosis inhibitors, while *Tmem79* cKO mice display increased lipid droplet accumulation (Figure S2C), enrichment of ferroptosis-associated pathways in keratinocytes, and elevated lipid peroxidation. These findings are mechanistically consistent with prior studies implicating TMEM79 in lipid biology and oxidative stress regulation (17, 18, 20). Ferroptotic lipid peroxidation has been shown in other contexts to activate cGAS–STING and induce type I IFN signaling, which in turn can reinforce ferroptotic susceptibility, establishing a bidirectional loop (63–66). While our findings are consistent with this framework, they do not establish the hierarchy or causal relationship of ferroptosis and STING activation in vivo. Moreover, TMEM79-deficient keratinocytes do not autonomously mount a clear STING/type I IFN response in vitro. Ferroptotic stress alone may be insufficient to activate the full STING-dependent inflammatory program outside the tissue environment. Instead, TMEM79 loss may create a permissive epithelial stress state that, together with barrier disruption, accumulated cell damage, microbial or environmental exposure, and immune cell interactions, engages STING-dependent type I IFN signaling in vivo.

Together, our findings support a model in which keratinocyte-intrinsic TMEM79 deficiency compromises keratinocyte differentiation and barrier integrity, induces ferroptotic lipid stress, and creates a tissue environment permissive for STING-dependent type I IFN signaling. This early barrier defect and inflammatory milieu, together with previously reported skin microbial dysbiosis (19), facilitates type 17 inflammation, which drives subsequent epithelial hyperplasia and chronic skin pathology. A limitation of the present study is that the causal relationship between ferroptosis and STING activation could not be directly tested at early postnatal time points. Nonetheless, our findings indicate that TMEM79-dependent epithelial homeostasis and innate immune sensing are determinants of inflammatory skin disease, and suggest that restoring TMEM79 function or epithelial differentiation, or targeting downstream ferroptotic lipid peroxidation and STING activation, may help prevent the transition from epithelial stress to chronic immune dysregulation. Given the emerging links among barrier dysfunction, ferroptosis, and innate immunity across epithelial tissues, this TMEM79-associated epithelial stress pathway may represent a generalizable mechanism by which epithelial barrier defects evolve into chronic inflammatory disease.

## Materials and methods

All materials and methods used in this study are detailed in the SI Appendix, Materials and Methods, including mice, quantitative RT-PCR analysis, histology and immunostaining, TEWL, epidermal and dermal sheet separation, preparation of single-cell suspensions from skin tissue, scRNA-seq, data processing, quality control, and annotation, differential gene expression analysis, bulk RNA-seq analysis of mouse epidermal sheets, flow cytometry, TEM, enzyme-linked immunosorbent assay (ELISA), cell culture and chemicals, generation of N/TERT-1 TMEM79 knockout lines, 2D differentiation of N/TERT-1 cells, TEER assay, transcriptomic analysis of human keratinocytes, LDH cytotoxicity assay, CCK-8 assay, lipid peroxidation assay (BODIPY-C11), clonogenic assay, EdU proliferation assay, and statistics. All animal experiments were approved by the Institutional Animal Care and Use Committee (IACUC) of Boston Children's Hospital (Protocol No. 00002200) and conducted in accordance with institutional guidelines and regulations.

## Supplementary Material

pgag361_Supplementary_Data

## Data Availability

The bulk and single-cell RNA-seq data generated in this study have been deposited in the NCBI Sequence Read Archive (SRA) under accession numbers PRJNA1470580, PRJNA1470727, PRJNA1535815, and PRJNA1535820.
