# Supplementary material for: Keratinocyte TMEM79 protects early life epidermal homeostasis and restrains STING-dependent skin inflammation
Source: PNAS Nexus. 2026 Oct 28;5(11):pgag361. doi: 10.1093/pnasnexus/pgag361 (PMC13732302; doi:10.1093/pnasnexus/pgag361)
Supplement: Supplementary file 1 [file pgag361_supplementary_data.pdf]

PNAS nexus

## Supplementary Information for

**Keratinocyte TMEM79 protects early life epidermal homeostasis and restrains STING-dependent skin inflammation**

Mengxu Ge[1**], Danilo Predes[1**], Andrew Kwong[2,3], Eunice To[1], Maorong Chen[1,4], Aakanksha Jain[1], Wenyi Wei[5], Jose Ordovas-Montanes[2,6-9], Manuel Leyva-Castillo[10], Isaac M. Chiu[11,12*], Xi He[1*]

**M.G. and D.P. contributed equally to this work.

*Corresponding authors: Isaac M. Chiu and Xi He

Email: isaac_chiu@hms.harvard.edu and Xi.He@childrens.harvard.edu

**SI Appendix, Materials and Methods**

**Mice**

K14-Cre (Strain No. 018964), *Sting1* KO (Strain No. 025805), *Ifnar1* KO (Strain No. 028288), *Mavs* KO (Strain No. 008634), Langerin-DTA (Strain No. 017949), TCF/Lef:H2B-GFP (Strain No. 013752), and wild-type C57BL/6J mice were purchased from The Jackson Laboratory. *Il-17a* KO mice have been previously described (1, 2). Mice were maintained under specific pathogen–free (SPF) conditions with controlled temperature, humidity, and a 12-hour light/dark cycle. All animal experiments were approved by the Institutional Animal Care and Use Committee (IACUC) of Boston Children's Hospital (Protocol No. 00002200) and conducted in accordance with institutional guidelines and regulations. Both male and female mice were used in most experiments, and no significant sex-dependent differences were observed. Female mice were used for the single-cell RNA sequencing (scRNA-seq) and bulk RNA-seq analyses of epidermal sheets.

**Quantitative RT-PCR Analysis**

Total RNA was isolated from mouse dorsal skin, ear skin, epidermal sheets, dermal sheets, or human keratinocytes as described in the Results section, using the RNeasy Plus Mini Kit (Cat. No. 74316, Qiagen). Two micrograms of total RNA were reverse-transcribed into cDNA using M-MLV Reverse Transcriptase (Cat. No. M1705, Promega) with RNasin® Ribonuclease Inhibitor (Cat. No. N2515, Promega), dNTP Mix (Cat. No. U1515, Promega), and Oligo(dT) primers (Cat. No. C1101, Promega). RT-PCR was performed on an Applied Biosystems 7500 Fast Real-Time PCR System using Fast SYBR Green Master Mix (Cat. No. 4385612, Applied Biosystems). Gene expression levels were normalized to *Gapdh* as an internal control. The RT-PCR primers used are listed in Table S1.

**Histology and Immunostaining**

Skin samples were fixed in 4% paraformaldehyde (PFA), embedded in paraffin, and sectioned at 4–5 μm thickness. After deparaffinization and rehydration, sections were stained with hematoxylin and eosin (H&E) or toluidine blue. All histological processing and staining were performed at the Rodent Histopathology Core, Harvard Medical School. For immunostaining, mouse dorsal skin samples were embedded in Tissue-Tek O.C.T compound and snap-frozen on dry

ice with 100% ethanol. Frozen sections (12–15 μm) were cut using a Leica CM1950 cryostat and mounted on Superfrost Plus slides (Cat. No. 48311-703, VWR). Sections were air-dried for 30 minutes at room temperature and fixed in 4% PFA for 15 minutes. After washing with PBS, sections were incubated in 0.1 M glycine for 10 minutes to quench residual PFA, followed by additional PBS washes. Samples were then blocked in PBS containing 5% normal goat serum, 2% bovine serum albumin (BSA), and 0.2% Triton X-100 for 1 hour at room temperature. Primary antibodies were diluted in blocking buffer and applied overnight at 4 °C. After washing, sections were incubated with Alexa Fluor–conjugated secondary antibodies (1:1000 dilution in blocking solution) and DAPI (0.2 μg/mL) for 1 hour at room temperature in the dark. Finally, slides were mounted using ProLong™ Diamond Antifade Mountant with DAPI (Cat. No. P36962, Thermo Fisher Scientific) and covered with glass coverslips to avoid air bubbles. Images were captured with a Zeiss LSM 700 confocal microscope or a Zeiss Axio Imager.Z2 microscope and subsequently processed using Fiji and Adobe Photoshop software. The primary antibodies used were as follows: K14 (1:500, Cat. No. ab7800, Abcam), K10 (1:1000, Cat. No. 905401, BioLegend), Ki67 (1:500, Cat. No. ab15580, Abcam), K6A (1:500, Cat. No. 905702, BioLegend), Myeloperoxidase (1:200, Cat. No. ab9535, Abcam), TCR γ/δ (1:200, Cat. No. 118101, BioLegend), and Langerin (1:200, Cat. No. DDX0362P-100, Novus Biologicals).

**Transepidermal Water Loss (TEWL)**

TEWL was measured to assess skin barrier function using a Cortex Technology USB DermaLab-TEWL X system (cyberDERM Inc.) following the manufacturer's instructions. Mice were briefly anesthetized with 4% isoflurane to minimize movement during measurement. TEWL values were recorded from the dorsal skin after shaving the mice 24 hours prior to the assay to avoid irritation. Measurements were taken at room temperature (approximately 22–24 °C) and constant humidity (40–60%). Three consecutive readings were obtained from each site, and the average value was used for analysis.

**Epidermal and Dermal Sheet Separation**

Epidermal and dermal sheets were separated from mouse ear or dorsal skin. For dorsal skin, subcutaneous adipose tissue and muscle were carefully removed using a sterile scalpel. The skin was then floated dermal side down in Dispase II solution (4 mg/mL, Cat. No. 17105041, Gibco) at 37 °C for 30 minutes. For ear skin, samples were first placed on ice for 5 minutes to facilitate separation of the dorsal and ventral sides using fine forceps, then transferred to Dispase II solution (4 mg/mL) and incubated at 37 °C for 30 minutes. Following incubation, the epidermis was gently peeled away from the dermis using fine forceps. Separated epidermal and dermal sheets were immediately rinsed in cold PBS and subjected to RNA extraction or flow cytometry.

**Preparation of Single-Cell Suspensions from Skin Tissue**

For 4-week-old mice, dorsal skin was cleared of subcutaneous adipose tissue and muscle, minced with scissors, and digested in complete RPMI medium supplemented with 0.2 mg/mL Liberase TL (Cat. No. 05401020001, Roche) and 0.1 mg/mL DNase I (Cat. No. 10104159001, Sigma) for 90 minutes at 37°C with shaking at 185 rpm, as previously described (3, 4). Following digestion, single-cell suspensions were prepared, and either live cells or CD45+ cells were sorted for subsequent single-cell RNA-seq library preparation. Epithelial cells were poorly recovered by this protocol. To improve epithelial resolution, P6 skin was prepared as separate epidermal and dermal single-cell suspensions (5). Briefly, dorsal skin was separated into epidermal and dermal sheets as described above. Dermal sheets were digested in 0.2 mg/mL Liberase TL and 0.1 mg/mL DNase I for 20 minutes at 37°C with shaking at 185 rpm, whereas epidermal sheets were digested in 0.25% Trypsin-EDTA for 10 minutes under the same conditions. The resulting cell suspensions were filtered, and live cells from the epidermal and dermal fractions were sorted and pooled at a 1:1 ratio to achieve a final concentration of 1,500 cells/μL.

## Single-Cell RNA Sequencing

Cell suspensions were processed into single-cell libraries using the Chromium Next GEM Single Cell 3' Reagent Kits v3.1 (10X Genomics). Libraries were sequenced on an Illumina NovaSeq 6000 platform using a NovaSeq S1 100-cycle kit at the Harvard Medical School Biopolymers Facility (Boston, MA, USA) and Novogene (Sacramento, CA, USA).

## Data Processing, Quality Control, and Annotation

A custom genome combining the mouse genome reference (mm10-2020-A) with the coding sequences of eGFP and tdTomato, inserted in the establishment of *Tmem79 fl/fl* mice (6), was created using the CellRanger mkref function. After sequencing, raw BCL files were demultiplexed using the CellRanger mkfastq function, and FASTQ files were aligned to this custom reference using CellRanger count (v7.1.0). Downstream analysis was performed using Seurat (v5.1.0) (7) with the 4W and P6 samples analyzed as separate datasets. An initial broad quality control filter was performed based on Unique Molecular Identifier (UMI) count (<25000), gene detection (<5500) and percentage of mitochondrial genes (<10%). Cells were normalized using SCTransform (v0.4.1) after which principal component analysis, Louvain clustering and UMAP embedding were conducted. A range of PCs and clustering resolutions were tested to select a final resolution. To identify cell states, iterative clustering was performed on individual cell types as previously described (8). At each level of annotation, doublets were identified based on co-expression of multiple lineage markers.

## Differential Gene Expression Analysis

Single-cell differential gene expression analysis, as necessary for identifying specific markers for cell annotation, was performed using a Wilcoxon rank sum test through the Seurat FindMarkers function. Genes were considered significant if they were expressed in a minimum of 20% of cells, exhibited a log-fold change of at least 0.25, and had an adjusted p value < 0.05.

Pseudobulk differential gene expression analysis was utilized when comparing across murine biological replicates. Within each combination of cell type and mouse, the gene expression of individual cells was aggregated using the Seurat AggregateExpression function. Differential gene expression analysis was then performed using the DESeq2 package (9). Genes with an absolute fold change > 1.2 and an adjusted p value < 0.05 were considered significantly differentially expressed. Gene Ontology (GO) and KEGG pathway enrichment analyses were performed separately on upregulated and downregulated gene sets using g:Profiler and Metascape (10).

## Bulk RNA-seq Analysis of Mouse Epidermal Sheets

Total RNA was extracted from epidermal sheets isolated from the dorsal skin of P1, P3, and P6 mice, and from the ear skin of P14 mice, using the RNeasy Plus Mini Kit (Cat. No. 74316; Qiagen) according to the manufacturer’s instructions. Ear skin was used for P14 mice because the dorsal skin at this stage, due to extensive hair follicle development, yields lower-quality epidermal sheets and suboptimal RNA integrity. RNA samples were then sent to Novogene (Sacramento, CA, USA) for library preparation and sequencing. Differential gene expression analysis was performed, and genes showing significant expression changes (false discovery rate [FDR] < 0.05 and |fold change| > 1.5) were selected for further analysis. Gene Ontology (GO) enrichment analyses were performed separately on upregulated and downregulated gene sets using g:Profiler and Metascape (10).

## Flow Cytometry

Dorsal skin, with subcutaneous adipose tissue and muscle removed, was minced into small pieces and incubated in 0.2 mg/mL Liberase TL (Roche) and 0.1 mg/mL DNase I (Sigma) for 60 minutes at 37 °C with shaking at 185 rpm to generate single-cell suspensions. Following digestion, cells were sequentially filtered through 70 μm and 40 μm cell strainers, washed in PBS containing 2% FBS, and incubated with a live/dead viability dye for 15 minutes at room temperature.

Cells were then blocked with Fc receptor blocking solution for 10 minutes at 4 °C before staining with fluorophore-conjugated antibodies against CD45 (AF700, 1:100, Cat. No. 103128, BioLegend), CD3 (PerCP-Cy5.5, 1:100, Cat. No. 100218, BioLegend), γδ TCR (FITC, 1:100, Cat. No. 107504, BioLegend), CD11b (APC, 1:100, Cat. No. 101212, BioLegend), and Ly6G (PE, 1:100, Cat. No. 127608, BioLegend). After staining, cells were washed, resuspended in PBS containing 2% FBS, and analyzed on a BD LSR II flow cytometer. Data were analyzed using FlowJo software. For gating, single live cells were identified using forward and side scatter, with dead cells and doublets excluded from analysis. Dermal γδ T cells were gated as $CD45^+CD3^+TCR\gamma\delta^+$ cells, epidermal γδ T cells as $CD45^+CD3^{high}TCR\gamma\delta^{high}$ cells, and neutrophils as $CD45^+CD11b^+Ly6G^+$ cells.

**Transmission Electron Microscopy (TEM)**

TEM was performed at the Electron Microscopy Facility of Harvard Medical School. Skin samples were fixed in 2.5% glutaraldehyde and 2% paraformaldehyde in 0.1 M sodium cacodylate buffer (pH 7.4) (Cat. No. 15949, Electron Microscopy Sciences) and processed for Epon resin embedding. Ultrathin sections (50–80 nm) were examined using a JEOL 1200EX transmission electron microscope, and images were captured with an AMT 2k CCD camera.

**Enzyme-Linked Immunosorbent Assay (ELISA)**

Blood samples were collected from mice and allowed to clot at room temperature for 30 minutes. Serum was then separated by centrifugation at 2,000 × g for 10 minutes at 4°C and stored at −80°C until analysis. Serum IL-17A levels were measured as previously described (11, 12). Briefly, an IL-17A-specific ELISA was performed using a plate-bound IL-17A capture antibody (1 µg/mL, Cat. No. 559501, BD Pharmingen) and a biotinylated IL-17A detection antibody (0.5 µg/mL, Cat. No. 555067; BD Pharmingen).

**Cell Culture and Chemicals**

HEK293T cells were obtained from ATCC, and low-passage N/TERT-1 human keratinocytes were obtained from Dr. Matthew R. Ramsey at Brigham and Women's Hospital (Boston, MA, USA). N/TERT-1 cells were routinely maintained in KGM-Gold medium (Lonza, 00192060) supplemented with 0.3 mM $CaCl_2$ and growth factors according to the manufacturer's instructions. Cells were kept at 37 °C in a humidified incubator with 5% $CO_2$ and were passaged before reaching full confluency to preserve proliferative capacity and differentiation potential. For routine maintenance, cells were counted (Countess 3 FL, Invitrogen) and plated at low density to avoid differentiation.

Cells were subcultured using TrypLE Express (Gibco, 12605010) once they reached 35% confluence. Following detachment, TrypLE was neutralized by dilution with medium containing serum, and the cells were centrifuged at 290 × g for 5 min and reseeded at the desired density.

Cells were treated as described with the following compounds: Ferrostatin-1 (HY-100579, MCE), Liproxstatin-1 (HY-12726, MCE), Necrostatin-1s (17802, CST), Z-VAD(Ome)-FMK (HY-16658, MCE), RSL3 (HY-100218A, MCE).

**Generation of N/TERT-1 TMEM79 Knockout Lines**

CRISPR/Cas9-mediated knockout of TMEM79 in N/TERT-1 keratinocytes was performed using the lentiCRISPRv2 system (Addgene plasmid #52961) (13). Guide RNAs (gRNAs) targeting the human TMEM79 locus were designed using online CRISPR design tools and cloned into lentiCRISPRv2, which co-expresses SpCas9 and the gRNA. Lentiviral particles were produced in HEK293T cells by co-transfection with lentiCRISPRv2 (TMEM79 gRNA: GCCAGTCCCCCAGGGCAAGG, non-targeting gRNA: AAATGTGAGATCAGAGTAAT) (14), psPAX2, and VSVG plasmids. Viral supernatants were collected 72 h post-transfection, filtered (0.45 µm), and used for transduction.

For transduction, $3 \times 10^4$ N/TERT-1 cells were seeded per well in 6-well plates two days prior to infection, reaching 30% confluence at the time of transduction. On the day of transduction, cells were exposed overnight to a mixture of KGM-

Gold medium (Lonza, 00192060) and viral supernatant supplemented with 4 μg/mL polybrene. The following day, the viral medium was replaced with fresh KGM-Gold. At 48 h post-transduction, cells were subjected to selection with puromycin (1 μg/mL); non-transduced controls died within 3 days. Medium containing puromycin was replaced every second day until resistant cells expanded.

When cells reached 70% confluence, cells were trypsinized, and single-cell clones were established by limiting dilution. Expanded clones were screened by PCR amplification of the target locus, followed by ICE analysis (Synthego) and TOPO cloning with Sanger sequencing. Knockout status was confirmed by the presence of frameshift mutations leading to premature stop codons, and lastly by western blot (anti-TMEM79 Ab: SAB1305998; anti-GAPDH Ab: #2118). In total, three independent TMEM79 knockout clones and three control clones (derived from cells transduced with non-targeting gRNA) were established and used in downstream experiments.

**2D Differentiation of N/TERT-1 Cells**

For two-dimensional differentiation assays, N/TERT-1 control and TMEM79 knockout clones were cultured in parallel. Cells were seeded at approximately 70% confluency, and upon reaching full confluency, differentiation was induced by supplementing KGM-Gold medium (Lonza) with 1.2 mM $CaCl_2$, as previously described for calcium-induced keratinocyte differentiation (15, 16). The day of calcium addition was designated as Day 0 (D0), and cells were maintained under differentiation conditions for the indicated number of days, with medium refreshed every other day.

Differentiated cells were collected at specific time points for RNA extraction and protein analysis. RNA samples were used for gene expression profiling by RNA-seq or qPCR to assess differentiation and epidermal marker expression.

**Transepithelial Electrical Resistance (TEER) Assay**

Barrier function was assessed by transepithelial electrical resistance (TEER) in N/TERT-1 keratinocytes differentiated at the air–liquid interface (ALI), following Smits et al. (2017) with modifications (17). Cell culture inserts (VWR, 10769-238; 24-well, PET membrane, 0.4 μm pore size, 0.33 $cm^2$ growth area) were coated with rat tail collagen type I (Corning) diluted to 100 μg/mL in PBS (1:30) and incubated for 1 h at room temperature. Coated inserts were rinsed once with PBS and partially dried under sterile conditions before use. A total of $7 \times 10^4$ N/TERT-1 cells were seeded in 50-100 μL medium into the upper compartment of each insert, with 450 μL medium added to the lower compartment. After 2 days, when cells reached confluence, the culture medium was replaced with differentiation medium consisting of CnT-PR-3D (CELLnTEC) and DMEM (60:40, v/v) in both the upper (50 μL) and lower (450 μL) compartments. After 24 h in differentiation medium, the upper medium was removed to establish the air–liquid interface. This point was designated as day 0, and cells were subsequently maintained at ALI for 10 days, with medium in the lower compartment refreshed every other day.

TEER was measured at the indicated time points using an EVOM3 voltohmmeter (World Precision Instruments) with EndOhm chambers (ENDOHM-6G, for 24-well inserts). For each insert, raw resistance (Ω) was corrected by subtracting the resistance of a blank (cell-free insert) and normalized to the membrane growth area (0.33 $cm^2$) to yield TEER values in $\Omega \cdot cm^2$.

For statistical analysis, duplicate inserts were measured for each of the three control and three knockout clones. The experiment was repeated independently three times for each clone. Data are presented as mean ± SEM across biological replicates.

**Transcriptomic Analysis of Human Keratinocytes**

Total RNA was extracted from N/TERT-1 control and TMEM79 knockout clones (three independent clones per group) using the RNeasy Plus Mini Kit (Qiagen) according to the manufacturer's protocol. RNA quality and integrity were verified prior to library preparation and sequencing, which were performed by Novogene (Sacramento, CA, USA).

Raw sequencing reads were aligned to the human reference genome (hg38) using STAR (18). Gene-level counts were obtained and differential expression analysis was performed with DESeq2 (9), applying an adjusted p-value cutoff of 0.05 for significance. Gene Ontology (GO) enrichment analyses (19) were conducted separately on upregulated and downregulated gene sets using Enrichr (20, 21) and the Molecular Signatures Database (MSigDB) (22, 23).

**LDH Cytotoxicity Assay**

Cell cytotoxicity was assessed using the CyQUANT™ LDH Cytotoxicity Assay Kit (Thermo Fisher Scientific, C20300), following the manufacturer's instructions with minor adaptations (24). $2 \times 10^4$ N/TERT-1 cells were seeded under 2D conditions in 48-well plates. After 48 h, cells were treated with the indicated compounds, and culture supernatants were collected at the specified time points.

For each experiment, a background control (medium only) and a maximum LDH release control (cells treated with the provided lysis buffer) were included. Following collection, supernatants were incubated with the LDH reaction mixture according to the kit protocol, and absorbance was measured at 490 nm with a 680 nm reference wavelength using a microplate reader.

Cytotoxicity was calculated as the percentage of LDH release relative to the maximum LDH control, after subtraction of background values. Data were normalized to the lysis buffer control within each condition to account for baseline variability.

**CCK-8 Assay**

Cell viability was measured using the Cell Counting Kit-8 (CCK-8; HY-K0301, MCE) according to the manufacturer's instructions. N/TERT-1 cells were seeded in 96-well plates at a density of $1.25 \times 10^3$ cells per well in KGM-Gold medium (Lonza) and cultured under 2D conditions. Twenty-four hours after plating, cells were treated as indicated and subsequently incubated for the specified duration. At the indicated time point, 10 µL of CCK-8 reagent was added directly to each well and incubated for the time recommended by the manufacturer at 37 °C in 5% $CO_2$.

Absorbance was measured at 450 nm using a microplate reader. Background signal from blank wells containing medium and reagent but no cells was subtracted from all readings. Relative cell viability was expressed as absorbance normalized to the corresponding control condition.

**Lipid Peroxidation Assay (BODIPY C11)**

Lipid peroxidation was quantified using BODIPY™ C11 (581/591) (Invitrogen, D3861), which increases green fluorescence upon oxidation. N/TERT-1 cells ($3 \times 10^4$ cells/well) were plated in 12-well plates (standard growth medium) and treated as indicated; single-cell suspensions of mouse epidermal sheets (when used) were stained identically after preparation. Cells were incubated with BODIPY C11 (2 µM, 30 min, 37 °C, protected from light), washed once with PBS, and immediately analyzed by flow cytometry. Acquisition used 488 nm excitation with collection in the green (530/30 nm) channel; data were reported as green-channel MFI (Oxidized BODIPY-C11) (25, 26). Samples were processed promptly to minimize ex vivo oxidation.

**Clonogenic Assay**

N/TERT-1 cells were seeded at 200 cells per well in 12-well plates and allowed to adhere for 24 h. Where indicated, treatments were added after this 24 h period and cells were maintained for 7 days, with medium changes every 2 days.

Colonies were fixed in methanol and stained with 0.5% crystal violet. Colonies were counted manually under a stereomicroscope, and results were recorded as colonies per well (27).

**EdU Proliferation Assay**

N/TERT-1 cells were seeded on sterile glass coverslips placed in 24-well plates and cultured in triplicate. At 48 h after plating, cells were pulsed with EdU for 1 h in growth medium and then fixed with 4% paraformaldehyde and permeabilized according to the Click-iT EdU imaging kit instructions (Thermo Fisher Scientific, C10339). Nuclei were counterstained with Hoechst 33342. For each coverslip, fluorescence images were acquired using identical exposure settings. $EdU^+$ nuclei and total nuclei ($Hoechst^+$) were quantified, and proliferation was reported as % $EdU^+$ cells.

**Statistics**

Data were analyzed using Microsoft Excel and GraphPad Prism 10 software. For comparisons between two groups, data are presented as mean ± SEM, and statistical significance was determined using an unpaired two-tailed Student's *t*-test unless otherwise stated in the figure legends. For comparisons among more than two groups in mouse experiments, data are presented as mean ± SEM, and statistical significance was determined by ordinary one-way ANOVA followed by Tukey's multiple comparisons test. For human keratinocyte experiments, comparisons among multiple groups were analyzed by ordinary one-way ANOVA followed by Dunnett's multiple comparisons test. Data are presented as mean ± SD. Statistical significance is denoted as follows: *$p < 0.05$, **$p < 0.01$, ***$p < 0.001$, ns, not significant.

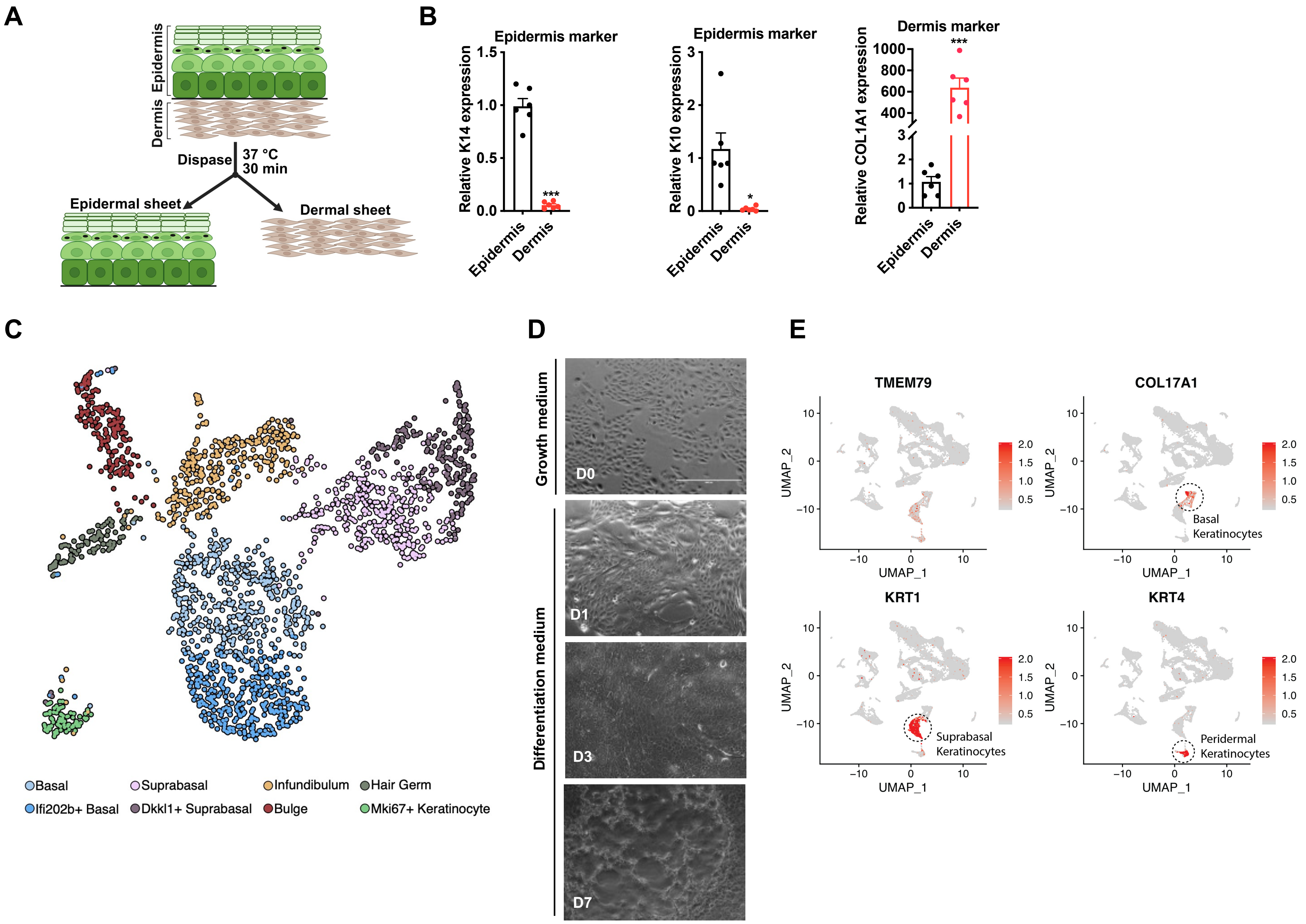


**Fig. S1. *TMEM79* expression increases with keratinocyte differentiation.**
**A.** Schematic illustration of epidermal and dermal separation to obtain epidermal sheets. The schematic was created using BioRender. **B.** qPCR analysis of *K14*, *K10*, and *Col1a1* expression in the epidermis and dermis. **C.** UMAP of adult dorsal skin in telogen from published scRNA-seq data. **D.** Images of primary human keratinocytes cultured in growth and differentiation media. **E.** UMAP feature plots of *TMEM79* and keratinocyte subtype markers in published human skin organoid scRNA-seq data.

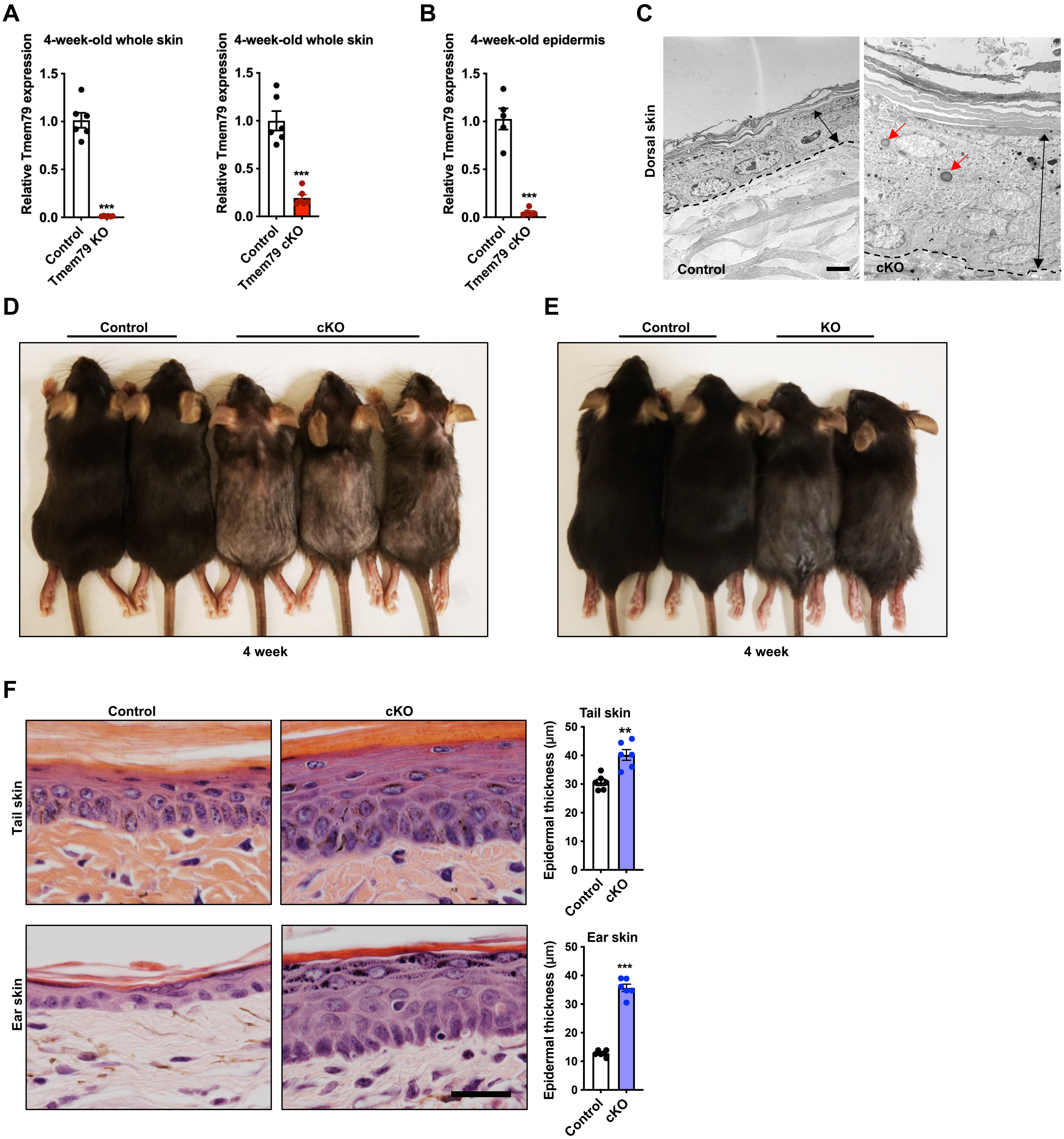


**Fig. S2. *Tmem79* loss leads to epidermal hyperplasia across multiple skin regions.**
**A.** qPCR analysis of *Tmem79* expression in whole skin across global and cKO models at 4 weeks of age. **B.** qPCR analysis of *Tmem79* expression in the epidermis of control and cKO mice at 4 weeks of age (n=5). **C.** TEM images of the epidermis from 4-week-old control and cKO mice. Double-headed arrows indicate epidermal thickness. Single-headed arrows point to lipid droplets. Black dashed lines mark the epidermal–dermal boundary. Scale bar = 2 μm. **D.** Representative photographs of 4-week-old control and cKO mice. **E.** Representative photographs of 4-week-old control and KO mice. **F.** H&E staining of tail and ear skin from 4-week-old control and cKO mice, with quantification of epidermal thickness. Scale bar = 25 μm.

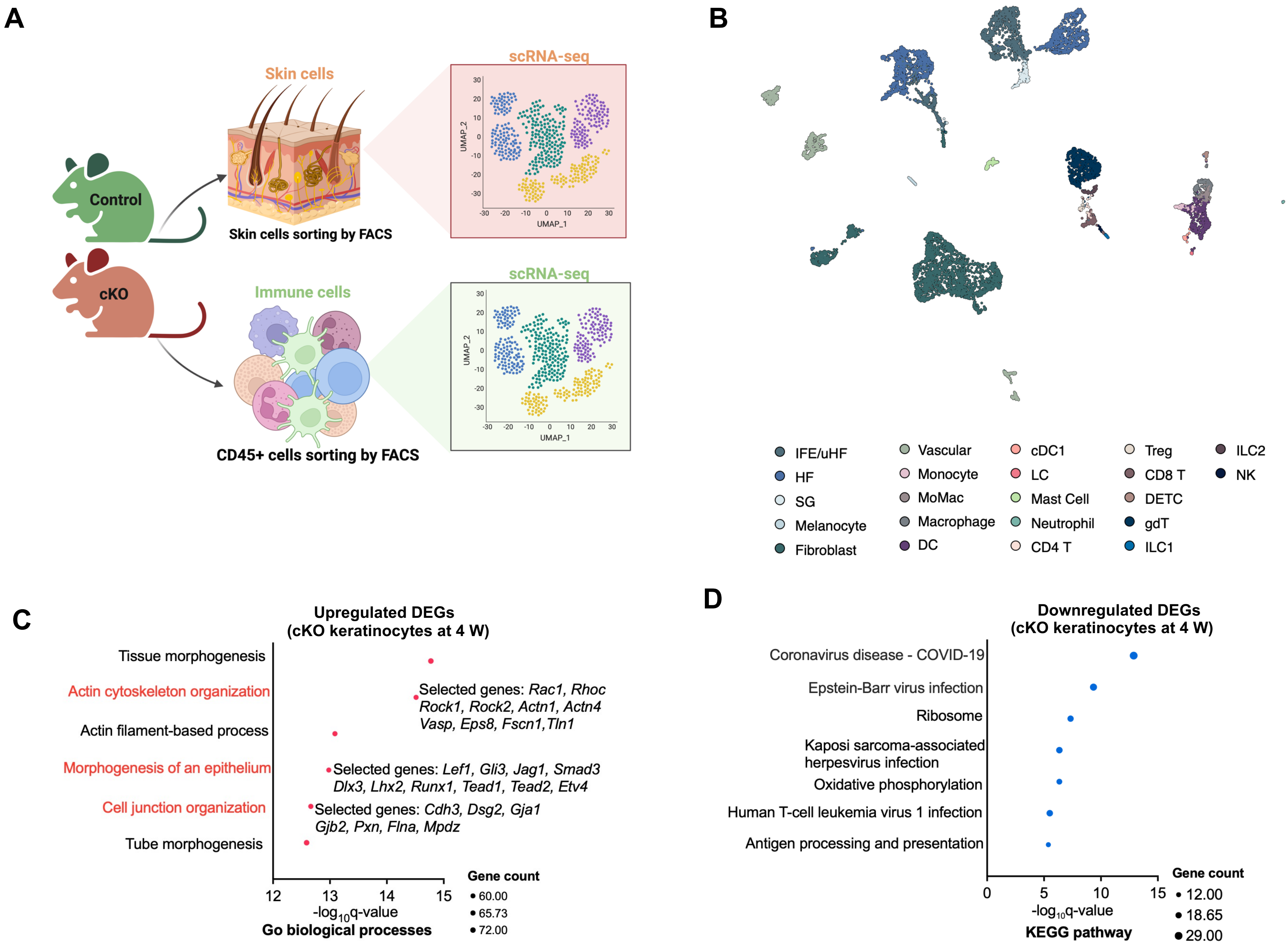


**Fig. S3. scRNA-seq analysis of 4-week-old *Tmem79* cKO mice.**
**A.** Schematic of the scRNA-seq strategy. **B.** UMAP of distinct cell types in whole skin identified by scRNA-seq. **C.** GO biological process analysis of upregulated DEGs in cKO keratinocytes, with selected enriched terms highlighted in red and associated genes indicated. **D.** KEGG pathway analysis of downregulated DEGs in cKO keratinocytes. Interfollicular epidermis/upper hair follicle (IFE/uHF), hair follicle (HF), sebaceous gland (SG), monocyte-derived macrophages (MoMac), dendritic cells (DC), conventional dendritic cell type 1 (cDC1), Langerhans cells (LC), regulatory T cells (Treg), dendritic epidermal T cells (DETC), γδ T cells (gdT), group 1 innate lymphoid cells (ILC1), group 2 innate lymphoid cells (ILC2), natural killer cells (NK).

Indels in human *TMEM79* locus

```
                                                protospacer
WT     5'.. GTCTAGACAGCACAGTAAGTGAGGCTGCCACC-TTGCCCTGGGGGACTGGCCCTCAGCCCAGTGCTCCGTTCCCGGATCCCC ..3'

KO #1 | +1  GTCTAGACAGCACAGTAAGTGAGGCTGCCACCTTTGCCCTGGGGGACTGGCCCTCAGCCCAGTGCTCCGTTCCCGGATCCCC
      | -20 GTCTAGACAGCACAGTAAGTGAGGCTGCCA---------------------CCTCAGCCCAGTGCTCCGTTCCCGGATCCCC

                                                protospacer
WT     5'.. GTCTAGACAGCACAGTAAGTGAGGCTGCCACC-TTGCCCTGGGGGACTGGCCCTCAGCCCAGTGCTCCGTTCCCGGATCCCC ..3'

KO #2 | +1  GTCTAGACAGCACAGTAAGTGAGGCTGCCACCTTTGCCCTGGGGGACTGGCCCTCAGCCCAGTGCTCCGTTCCCGGATCCCC
      | +1  GTCTAGACAGCACAGTAAGTGAGGCTGCCACCTTTGCCCTGGGGGACTGGCCCTCAGCCCAGTGCTCCGTTCCCGGATCCCC

                                                protospacer
WT     5'.. GTCTAGACAGCACAGTAAGTGAGGCTGCCACC--TTGCCCTGGGGGACTGGCCCTCAGCCCAGTGCTCCGTTCCCGGATCCCC..3'

KO #3 | +2  GTCTAGACAGCACAGTAAGTGAGGCTGCCACCTTTTGCCCTGGGGGACTGGCCCTCAGCCCAGTGCTCCGTTCCCGGATCCCC
      | +1  GTCTAGACAGCACAGTAAGTGAGGCTGCCACC-TTTGCCCTGGGGGACTGGCCCTCAGCCCAGTGCTCCGTTCCCGGATCCCC
```

**Fig. S4.** Genomic DNA sequencing demonstrating indel (frameshift) mutations in *TMEM79* alleles in the three TMEM79 KO human keratinocyte clones used in this study.

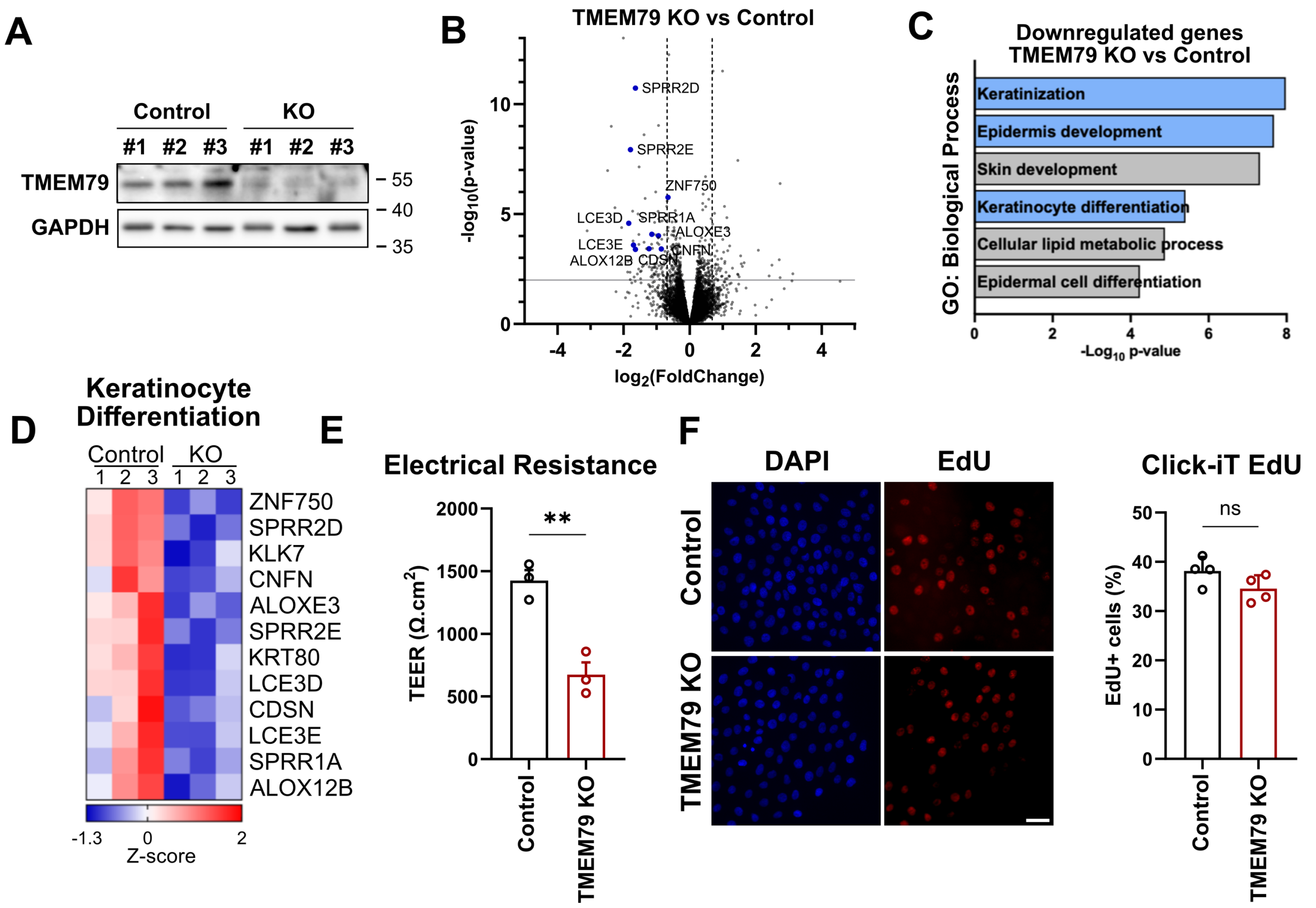


**Fig. S5. TMEM79 deficiency impairs human keratinocyte differentiation and barrier formation.**

**A.** Immunoblot confirming loss of TMEM79 protein in N/TERT-1 keratinocyte knockout clones. **B.** Volcano plot of differentially expressed genes in control and TMEM79-deficient N/TERT-1 cells after 3 days of calcium-induced differentiation. Downregulated keratinocyte differentiation genes are highlighted in blue. **C.** Gene Ontology (GO) enrichment analysis of downregulated genes in TMEM79-deficient cells, revealing significant enrichment of pathways related to keratinocyte differentiation and epidermal development. **D.** Heatmap showing reduced expression of selected keratinocyte differentiation markers in TMEM79-deficient cells compared to controls. **E.** Transepithelial electrical resistance (TEER) measurements of three-dimensional human epidermal equivalents generated from control and TMEM79-deficient N/TERT-1 cells and cultured under air–liquid interface (ALI) conditions for 10 days. Data are shown as mean ± SEM (n = 3 clones); $^{**}p < 0.01$. **F.** Representative images and quantification of Click-iT EdU incorporation showing comparable percentages of EdU-positive cells between control and TMEM79-deficient N/TERT-1 cells. Scale bar = 40 µm. Data are shown as mean ± SD (n = 4 independent replicates using clone WT #1 and clone KO #1); ns, not significant.

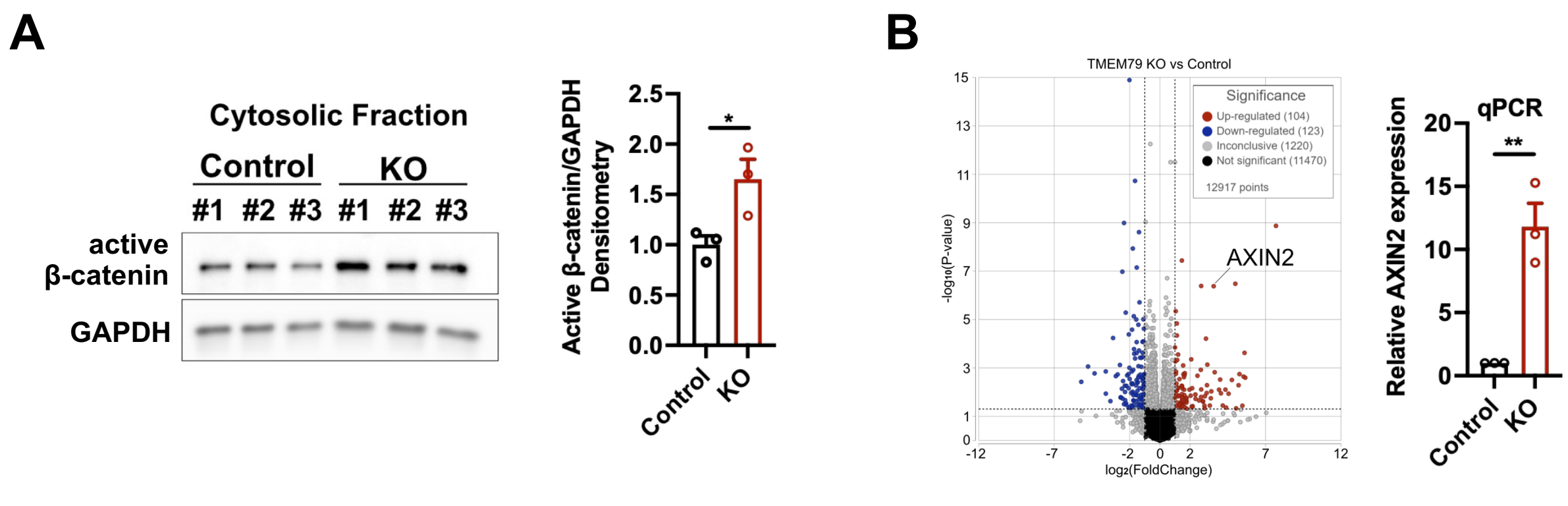


**Fig. S6. Wnt activation is associated with increased inflammatory gene expression in keratinocytes.**

**A.** Western blotting for cytosolic β-catenin and densitometry quantification in control and KO clones. **B.** Volcano plot analysis and qPCR validation of *AXIN2* expression in control and KO clones.

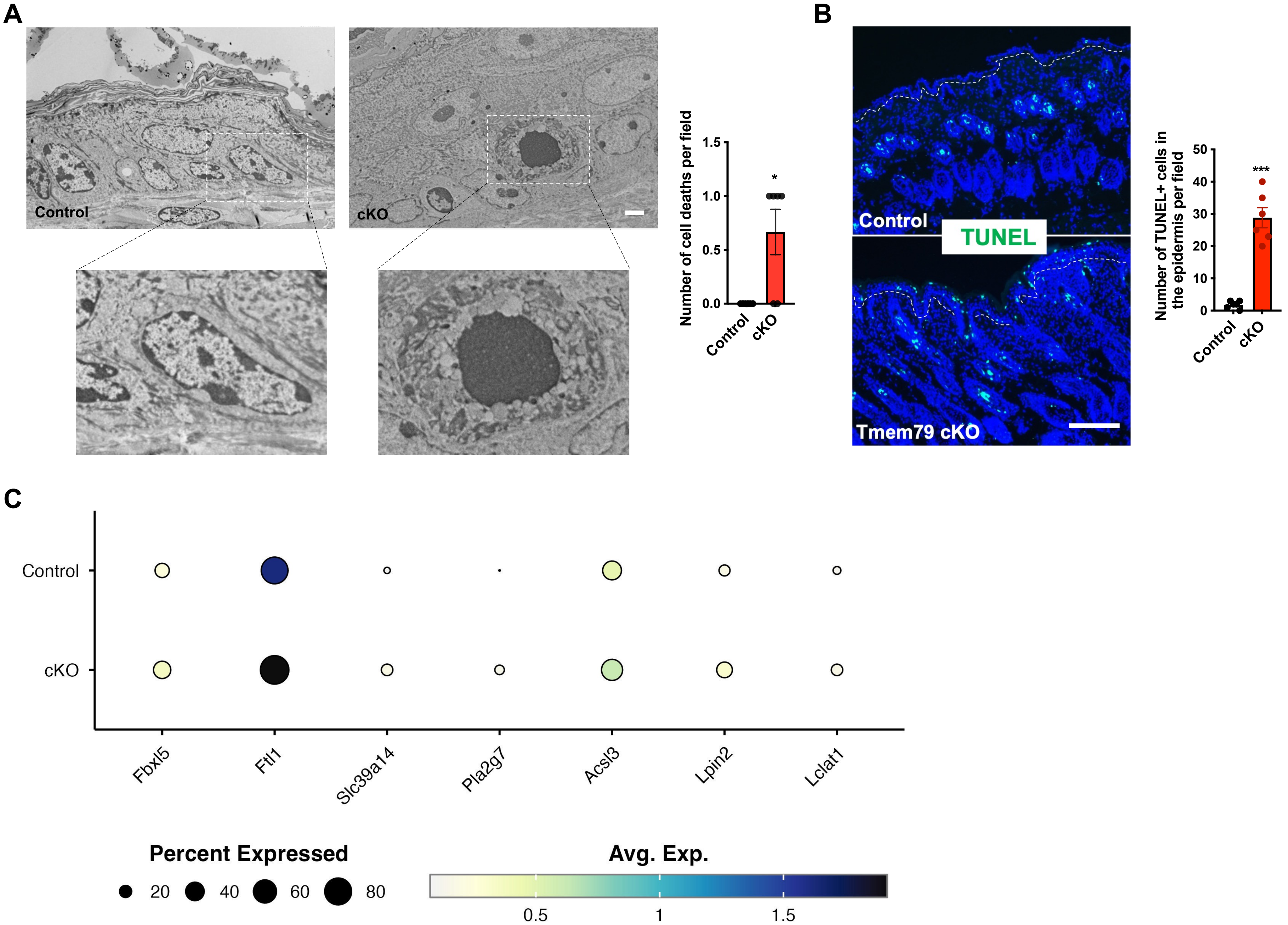


**Fig. S7.** ***Tmem79* deficiency promotes keratinocyte cell death and ferroptosis-associated transcriptional changes.** **A.** TEM images of control and cKO mice at 4 weeks reveal an increased number of dying cells in cKO mice. Scale bars, 2 μm. **B.** TUNEL staining of skin from control and cKO mice at 4 weeks, with quantification of TUNEL$^+$ cells in the epidermis. White dashed lines indicate the epidermal and dermal boundary. Scale bars, 100 μm. **C.** Dot plot showing the expression of ferroptosis- and lipid metabolism-related genes in keratinocytes from control and cKO mice at 4 weeks.

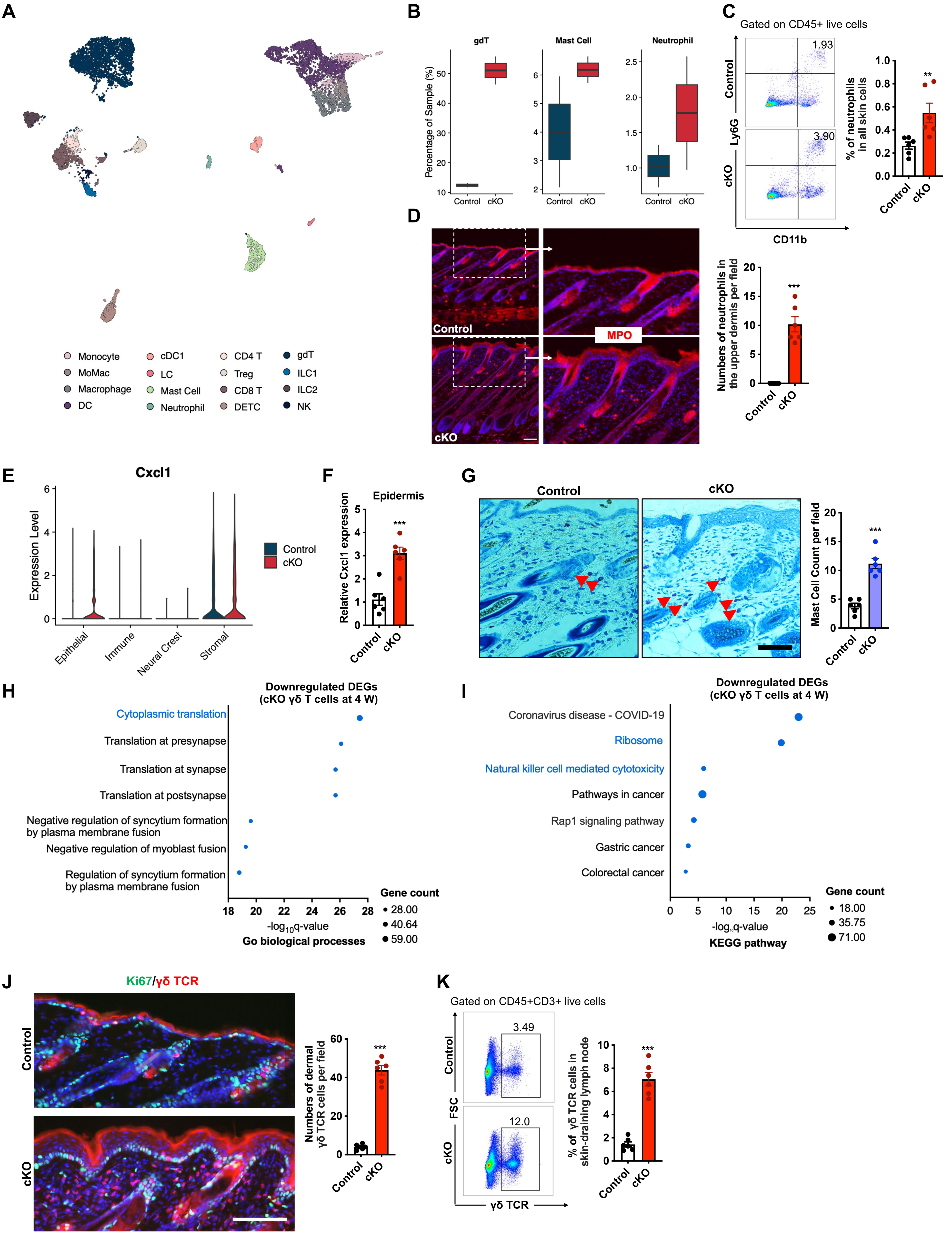
A
Monocyte
MoMac
Macrophage
DC
cDC1
LC
Mast Cell
Neutrophil
CD4 T
Treg
CD8 T
DETC
gdT
ILC1
ILC2
NK
B
gdT
Mast Cell
Neutrophil
Percentage of Sample (%)
Control
cKO
C
Gated on CD45+ live cells
Control
cKO
Ly6G
CD11b
1.93
3.90
% of neutrophils in all skin cells
**
D
Control
cKO
MPO
Numbers of neutrophils in the upper dermis per field
***
E
Cxcl1
Expression Level
Epithelial
Immune
Neural Crest
Stromal
Control
cKO
F
Epidermis
Relative Cxcl1 expression
***
G
Control
cKO
Mast Cell Count per field
***
H
Downregulated DEGs (cKO γδ T cells at 4 W)
Cytoplasmic translation
Translation at presynapse
Translation at synapse
Translation at postsynapse
Negative regulation of syncytium formation by plasma membrane fusion
Negative regulation of myoblast fusion
Regulation of syncytium formation by plasma membrane fusion
-log10q-value
Go biological processes
Gene count
28.00
40.64
59.00
I
Downregulated DEGs (cKO γδ T cells at 4 W)
Coronavirus disease - COVID-19
Ribosome
Natural killer cell mediated cytotoxicity
Pathways in cancer
Rap1 signaling pathway
Gastric cancer
Colorectal cancer
-log10q-value
KEGG pathway
Gene count
18.00
35.75
71.00
J
Ki67/γδ TCR
Control
cKO
Numbers of dermal γδ TCR cells per field
***
K
Gated on CD45+CD3+ live cells
Control
cKO
FSC
γδ TCR
3.49
12.0
% of γδ TCR cells in skin-draining lymph node
***

**Fig. S8. Immune cell profiles of inflamed skin in 4-week-old *Tmem79* cKO mice.**
**A.** UMAP of distinct immune cell types in $CD45^+$ FACS-sorted cells identified by scRNA-seq. **B.** Box plots show the percentage of γδ T cells (gdT), mast cells, and neutrophils among $CD45^+$ cells in skin from control and cKO mice. **C.** Representative FACS profiles and quantification of neutrophils in skin from control and cKO mice**. D.** Immunostaining of MPO in skin from control and cKO mice, with higher-magnification views of the upper skin. Quantification of $MPO^+$ cells in the upper dermis per field is shown. Scale bar = 100 μm. **E.** Violin plots show the distribution of *Cxcl1* expression across major cell compartments, including epithelial, immune, neural crest, and stromal cells, in control and cKO skin as determined by scRNA-seq. *Cxcl1* expression is predominantly increased in epithelial cells of cKO mice. **F.** qPCR analysis of *Cxcl1* expression in the epidermis of control and cKO mice. **G.** Toluidine blue staining and quantification of toluidine blue+ cells in control and cKO mice. Arrowheads indicate toluidine blue+ mast cells. Scale bar = 100 μm. **H.** GO biological process analysis of downregulated DEGs in cKO γδ T cells, highlighting selected enriched biological processes (blue). **I.** KEGG pathway enrichment analysis of downregulated DEGs in cKO γδ T cells. Selected enriched pathways are highlighted in blue. **J.** Immunostaining of Ki67 (green) and γδ TCR (red) in skin from control and cKO mice. Minimal overlap between Ki67 and γδ TCR signals is observed. Quantification of dermal γδ $TCR^+$ cells per field is shown. Scale bar = 100 μm. **K.** FACS profiles and quantification of γδ $TCR^+$ cells in skin-draining lymph nodes from control and cKO mice.

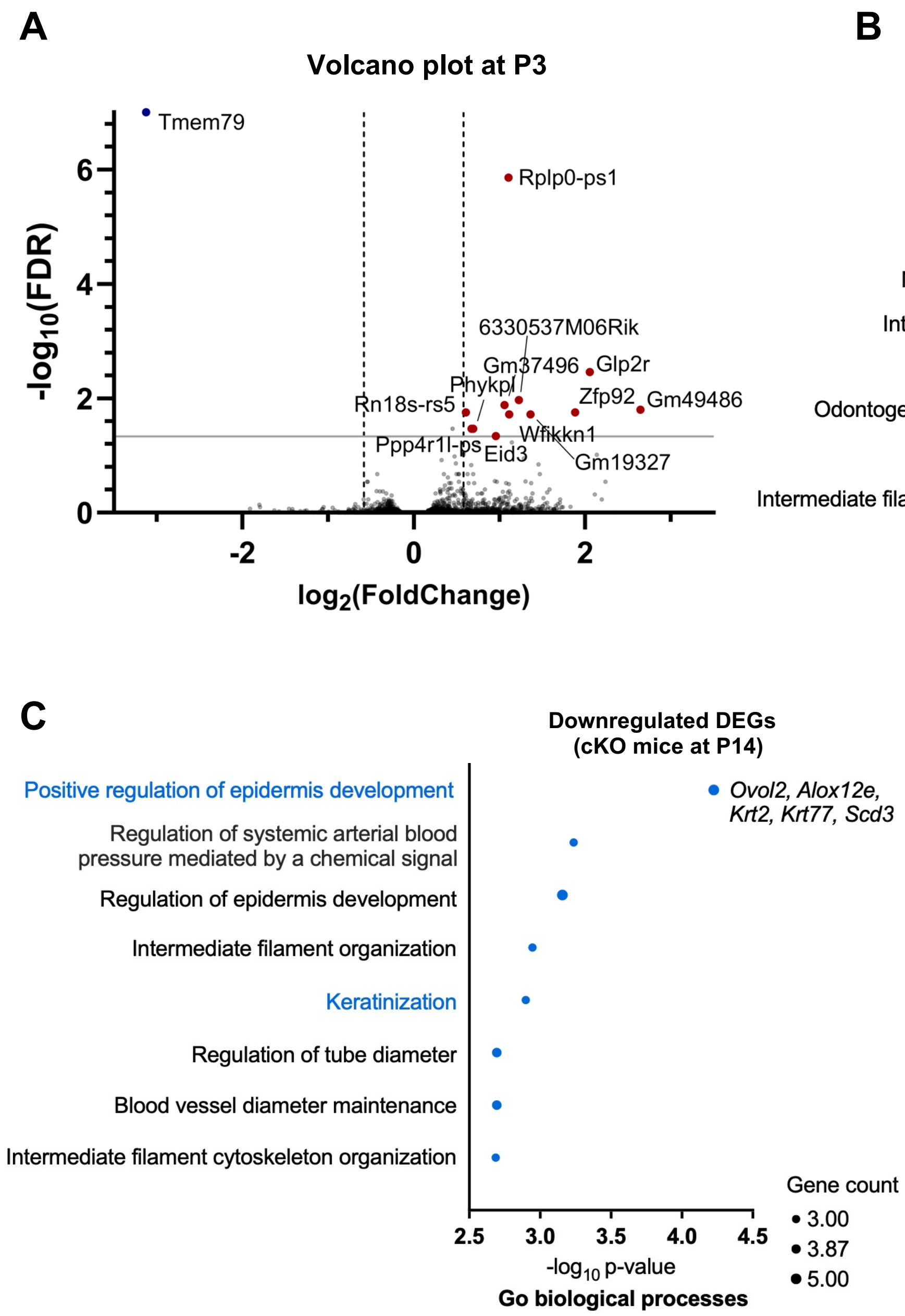


**Fig. S9. Impaired epidermal development and keratinization are enriched in bulk RNA-seq data from epidermal sheet samples of *Tmem79* cKO mice.**
**A.** Volcano plot showing DEGs in epidermal sheets from control and cKO mice at P3, with all significantly altered genes labeled. **B.** GO analysis of downregulated DEGs in cKO epidermis at P6, with epidermis development and keratinization highlighted in blue and the corresponding gene sets shown. **C.** GO analysis of downregulated DEGs in cKO epidermis at P14, with positive regulation of epidermis development and keratinization highlighted in blue and the associated gene sets shown.

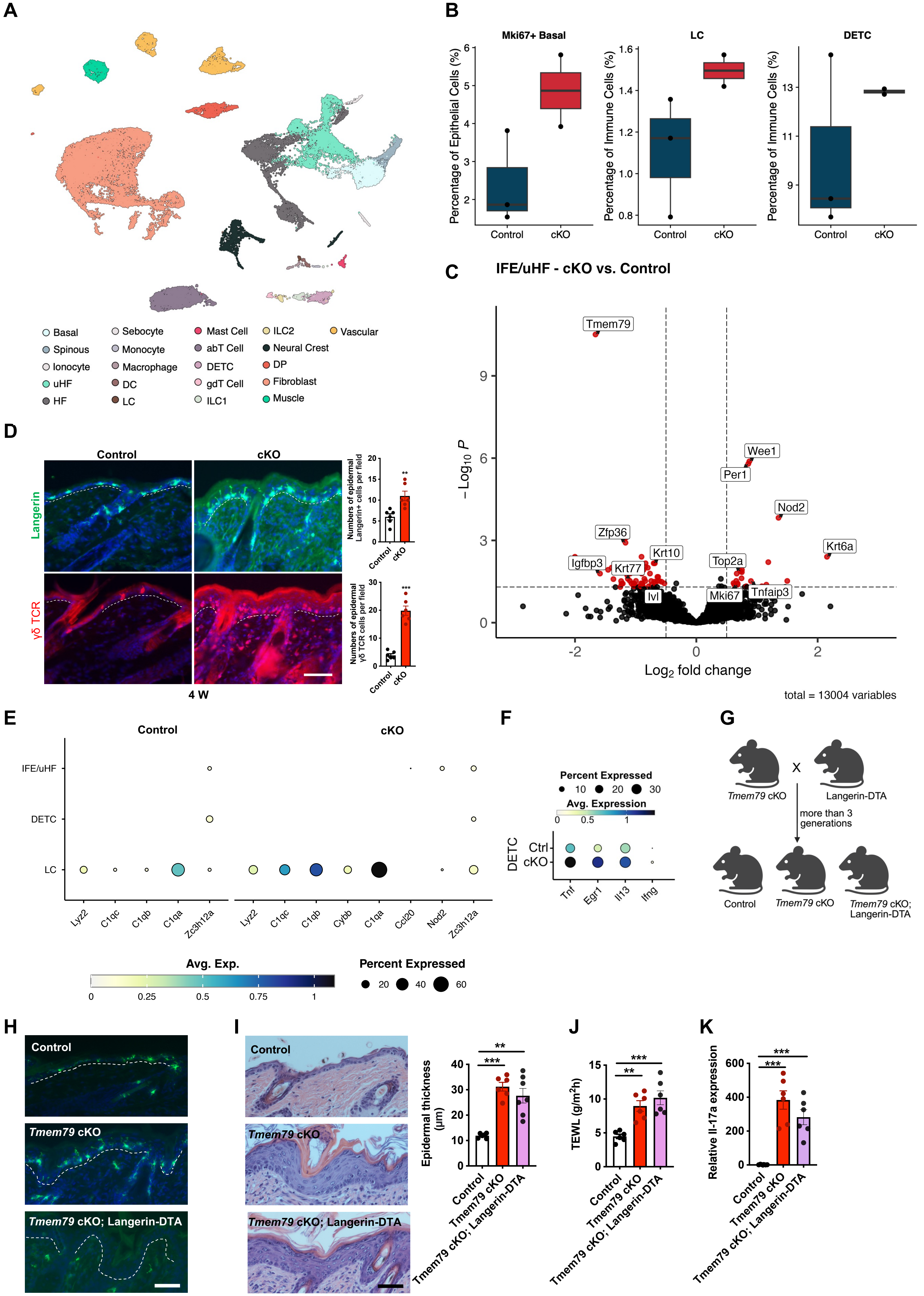
A
Basal
Spinous
Ionocyte
uHF
HF
Sebocyte
Monocyte
Macrophage
DC
LC
Mast Cell
abT Cell
DETC
gdT Cell
ILC1
ILC2
Neural Crest
DP
Fibroblast
Muscle
Vascular
B
Mki67+ Basal
LC
DETC
Percentage of Epithelial Cells (%)
Percentage of Immune Cells (%)
Control
cKO
C
IFE/uHF - cKO vs. Control
Tmem79
Wee1
Per1
Nod2
Zfp36
Krt10
Igfbp3
Krt77
Top2a
Krt6a
Ivl
Mki67
Tnfaip3
$-\mathrm{Log}_{10}\ P$
$\mathrm{Log}_2$ fold change
total = 13004 variables
D
Control
cKO
Langerin
γδ TCR
Numbers of epidermal Langerin+ cells per field
Numbers of epidermal γδ TCR cells per field
4 W
E
Control
cKO
IFE/uHF
DETC
LC
Lyz2
C1qc
C1qb
C1qa
Zc3h12a
Cybb
Ccl20
Nod2
Avg. Exp.
Percent Expressed
F
Percent Expressed
Avg. Expression
DETC
Ctrl
cKO
Tnf
Egr1
Il13
Ifng
G
Tmem79 cKO
X
Langerin-DTA
more than 3 generations
Control
Tmem79 cKO
Tmem79 cKO; Langerin-DTA
H
Control
Tmem79 cKO
Tmem79 cKO; Langerin-DTA
I
Control
Tmem79 cKO
Tmem79 cKO; Langerin-DTA
Epidermal thickness (μm)
J
TEWL (g/m²h)
K
Relative Il-17a expression

**Fig. S10. Langerhans cell ablation does not affect skin pathology in *Tmem79* cKO mice.**
**A.** UMAP of distinct cell types in whole skin identified by scRNA-seq at P6 (Control, n = 3; cKO, n = 2). **B.** Comparison of the proportions of specific epithelial and immune cell populations between P6 control and cKO mice. **C.** Volcano plot showing DEGs between P6 cKO and control keratinocytes, with selected genes highlighted. **D.** Immunostaining and quantification of Langerin and γδ TCR in control and cKO mice at 4 weeks. Scale bar = 50 μm. **E.** Dot plot depicting the expression of the upregulated inflammatory genes identified in bulk RNA-seq data from cKO epidermis at P6. **F.** Dot plot illustrating some inflammatory gene expression in P6 control and cKO DETCs. **G.** Schematic of the generation of *Tmem79* cKO and Langerin-DTA mice. **H.** Immunostaining for Langerin in 4-week-old control, *Tmem79* cKO, and *Tmem79* cKO; Langerin-DTA mice, showing complete depletion of LCs in *Tmem79* cKO; Langerin-DTA mice. Scale bar = 50 μm. **I–K.** H&E images and quantification of epidermal thickness (**I**), TEWL (**J**), and qPCR analysis of *Il17a* expression (**K**) in 4-week-old control (*Tmem79 fl/+* or *Tmem79 fl/fl*), *Tmem79* cKO, and *Tmem79* cKO; Langerin-DTA mice. Scale bar = 50 μm.

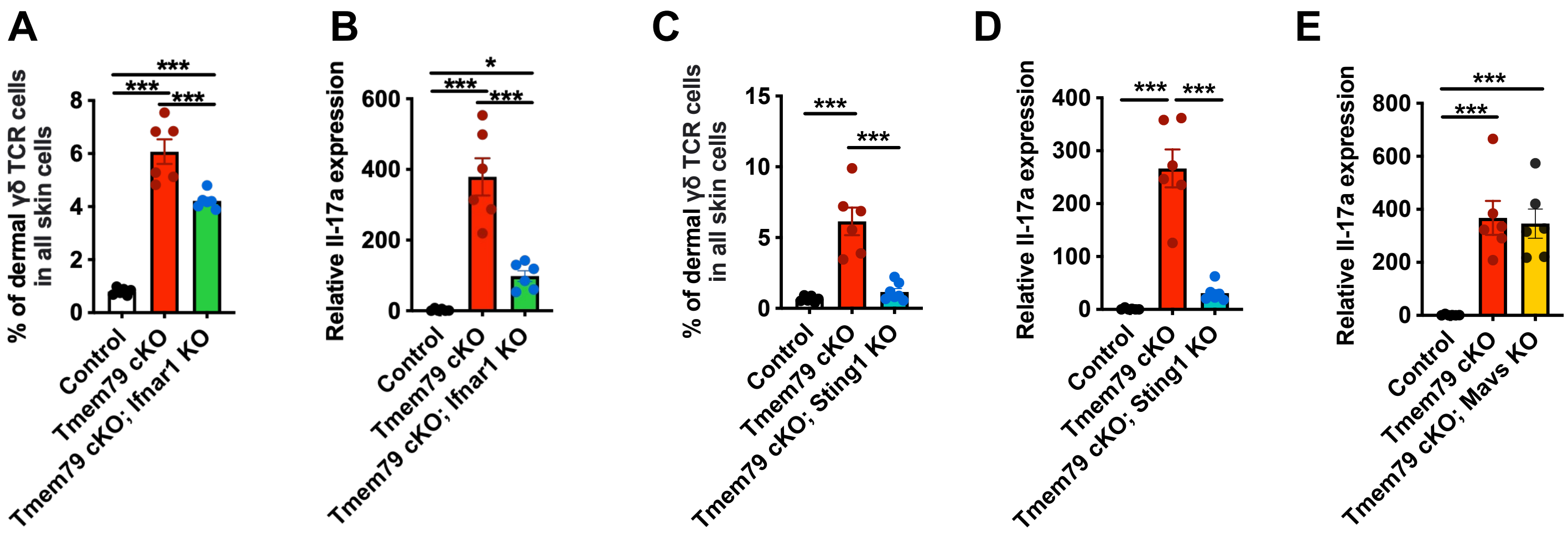


**Fig. S11. Early STING–dependent signaling drives type 17 responses in *Tmem79* cKO mice.**
**A-B**. Dermal γδ TCR⁺ cell abundance (**A**) and qPCR analysis of *Il17a* expression (**B**) in 4-week-old control, *Tmem79* cKO, and *Tmem79* cKO; *Ifnar1* KO mice. **C-D**. Dermal γδ TCR⁺ cell abundance (**C**) and qPCR analysis of *Il17a* expression (**D**) in 4-week-old control, *Tmem79* cKO, and *Tmem79* cKO; *Sting1* KO mice. **E**. qPCR analysis of *Il17a* expression in 4-week-old control, *Tmem79* cKO, and *Tmem79* cKO; *Mavs* KO mice.

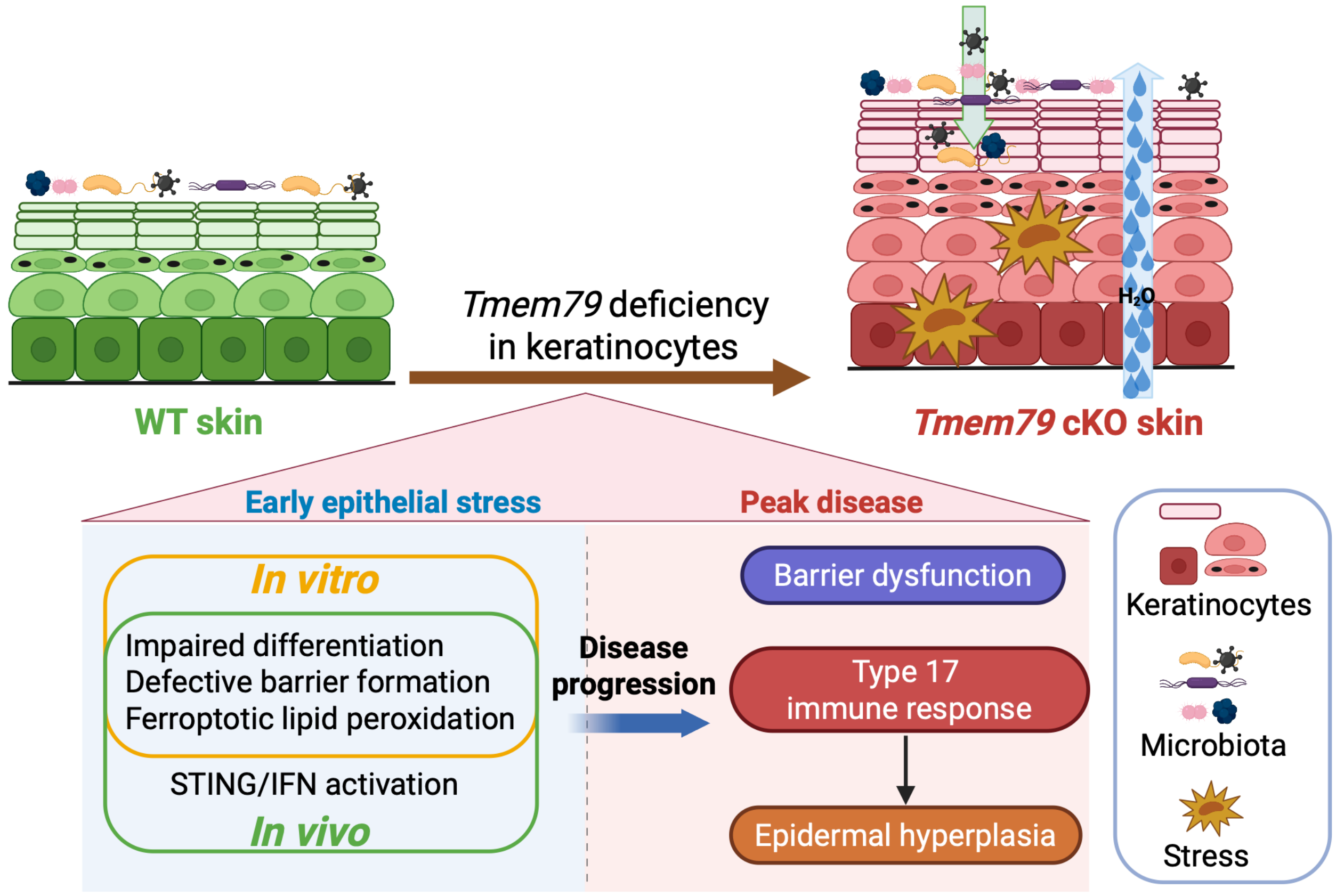


**Fig. S12. Model of TMEM79 function in epidermal homeostasis and skin inflammation.** In wild-type (WT) skin, intact epidermal barrier function maintains controlled transepidermal water loss (TEWL) and limits microbial penetration. Loss of *Tmem79* in keratinocytes initiates an early epithelial stress program characterized by impaired differentiation, defective barrier formation, increased lipid peroxidation, and activation of STING-dependent inflammatory signaling, including type I interferon responses. These early epithelial abnormalities promote disease progression, leading to barrier dysfunction, induction of a type 17 immune response, and epidermal hyperplasia, culminating in peak disease. The schematic model was created using BioRender.

**Table S1. List of RT-PCR primer sequences used in this study**

| Primer name | Sequence (5' to 3') |
|---|---|
| RT-Sting1-F | TGGGTACTTGCGGTTGATCT |
| RT-Sting1-R | ATGTACAGTCTTCGGCTCCC |
| RT-IFNAR1-F | AGGGAACAGCACATCTTCGT |
| RT-IFNAR1-R | CACATGTTCCCGTCTTGTCC |
| RT-Mavs-F | TATCCGAGACAACCACAGCA |
| RT-Mavs-R | TGTGAGGCAGGACAGGTAAG |
| RT-K14-F | ACAGCCCCTACTTCAAGACC |
| RT-K14-R | GCATTGTCGATCTGCAGGAG |
| RT-K10-F | TCAAACTACGAGCTGGAGGG |
| RT-K10-R | GTCAGTTGTCAGGGTGAGGA |
| RT-COL1A-F | TGGAGATGATGGGGAAGCTG |
| RT-COL1A-R | CCATCCAAACCACTGAAGCC |
| RT-PDGFA-F | CCATTAACCATGTGCCCGAG |
| RT-PDGFA-R | AGATCAGGAAGTTGGCCGAT |
| RT-Axin2-F | AAGAGAAGCGACCCAGTCAA |
| RT-Axin2-R | CTGCGATGCATCTCTCTCTG |
| RT-GAPDH-F | GAGAGGCCCTATCCCAACTC |
| RT-GAPDH-R | TCAAGAGAGTAGGGAGGGCT |
| RT-IL17A-F | TTTAACTCCCTTGGCGCAAAA |
| RT-IL17A-R | CTTTCCCTCCGCATTGACAC |
| RT-IL22-F | TCCAACTTCCAGCAGCCATA |
| RT-IL22-R | TAGCACTGACTCCTCGGAAC |
| RT-IL4-F | CAGCAACGAAGAACACCACA |
| RT-IL4-R | TCTGCAGCTCCATGAGAACA |
| RT-IL13-F | TCACACAAGACCAGACTCCC |
| RT-IL13-R | CTGGGTCCTGTAGATGGCAT |
| RT-IFNg-F | CCACGGCACAGTCATTGAAA |
| RT-IFNg-R | TGTCACCATCCTTTTGCCAG |
| RT-CXCL1-F | CTTGACCCTGAAGCTCCCTT |
| RT-CXCL1-R | GTTGTCAGAAGCCAGCGTTC |
| RT-CCL20-F | GAACTGGGTGAAAAGGGCTG |
| RT-CCL20-R | TCAACCCCAGCTGTGATCAT |
| RT-Ifi202b-F | AAGAAAGGCTGGTTGATGGAGAG |
| RT-Ifi202b-R | GGTCAGTTGTCCAGATACCACC |
| RT-Oasl2-F | CCAAAACGAGGTCGTCAGGAAC |
| RT-Oasl2-R | AGCCACCTGTTCCCATCCCTTT |
| RT-USP18-F | GGAACCTGACTAAGGACCAGATC |
| RT-USP18-R | GAGAGTGTGAGCAGTTTGCTCC |
| RT-Ifit1-F | ACAGCAACCATGGGAGAGAA |
| RT-Ifit1-R | GAACTGGACCTGCTCTGAGA |
| RT-Aim2-F | AGGCTGCTACAGAAGTCTGTCC |
| RT-Aim2-R | TCAGCACCGTGACAACAAGTGG |
| RT-Ddx60-F | AACTGTTCCTGCCTGCTTTG |
| RT-Ddx60-R | TCCCAGATTTCCGCTCCAAT |
| RT-Xaf1-F | CTGCGCTTCATAGTCCTTTGCC |
| RT-Xaf1-R | AGGGTGCTGTTGGCTTTCCTTG |
| RT-Oas1a-F | GAGGTGGAGTTTGATGTGCTGC |
| RT-Oas1a-R | GTGAAGCAGGTAGAGAACTCGC |

| | |
|---|---|
| RT-Tmem79-F | CATACGCCTTCCTGCCTTTC |
| RT-Tmem79-R | TACACAAGGAGCCCGAGTAC |
| RT-hTMEM79-F | CTGCTCACTGGTCTCTTTGC |
| RT-hTMEM79-R | GGTTCCACATCAGCATCGAC |
| RT-hAXIN2-F | CCACACCCTTCTCCAATCCA |
| RT-hAXIN2-R | CCTTCAGCATCCTCCGGTAT |
| RT-hGAPDH-F | CACCACACTGAATCTCCCCT |
| RT-hGAPDH-R | TGGTTGAGCACAGGGTACTT |
| RT-hK14-F | TCCTCTGGATCGCAGTCATC |
| RT-hK14-R | CTCAGTTCTTGGTGCGAAGG |
| RT-hK10-F | GGCCTTGAAACAATCCCTGG |
| RT-hK10-R | TGGGCCTGAATCTGTGAGAG |
| RT-hFLG-F | ATCCTCTCACCGCGATACAG |
| RT-hFLG-R | TGACCATGTTCCTTAGCGGT |
| RT-hLOR-F | CTCCTCACTCACCCTTCCTG |
| RT-hLOR-R | GCCAGAGGTCTTCACGCA |
| RT-hIVL-F | AGCCTTACTGTGAGTCTGGT |
| RT-hIVL-R | TGGGTATTGACTGGAGGAGG |

Note: Primer names prefixed with ‘h’ (e.g., hTMEM79) indicate human primers, while those without ‘h’ correspond to mouse primers.

**References**


1. J. M. Leyva-Castillo *et al.*, Basophil-derived IL-4 promotes cutaneous Staphylococcus aureus infection. *JCI Insight* **6** (2021).
2. S. Nakae *et al.*, Antigen-specific T cell sensitization is impaired in IL-17-deficient mice, causing suppression of allergic cellular and humoral responses. *Immunity* **17**, 375-387 (2002).
3. J. M. Leyva-Castillo *et al.*, Mechanical Skin Injury Promotes Food Anaphylaxis by Driving Intestinal Mast Cell Expansion. *Immunity* **50**, 1262-1275 e1264 (2019).
4. S. Leisten *et al.*, Development of skin lesions in filaggrin-deficient mice is dependent on adaptive immunity. *J Allergy Clin Immunol* **131**, 1247-1250, 1250 e1241 (2013).
5. S. Joost *et al.*, The Molecular Anatomy of Mouse Skin during Hair Growth and Rest. *Cell Stem Cell* **26**, 441-457 e447 (2020).
6. J. J. Emrick *et al.*, Tissue-specific contributions of Tmem79 to atopic dermatitis and mast cell-mediated histaminergic itch. *Proc Natl Acad Sci U S A* **115**, E12091-E12100 (2018).
7. Y. Hao *et al.*, Dictionary learning for integrative, multimodal and scalable single-cell analysis. *Nat Biotechnol* **42**, 293-304 (2024).
8. H. B. Zheng *et al.*, Concerted changes in the pediatric single-cell intestinal ecosystem before and after anti-TNF blockade. *eLife* **12**, RP91792 (2023).
9. M. I. Love, W. Huber, S. Anders, Moderated estimation of fold change and dispersion for RNA-seq data with DESeq2. *Genome Biol* **15**, 550 (2014).
10. Y. Zhou *et al.*, Metascape provides a biologist-oriented resource for the analysis of systems-level datasets. *Nat Commun* **10**, 1523 (2019).
11. L. E. Harrington *et al.*, Interleukin 17-producing CD4+ effector T cells develop via a lineage distinct from the T helper type 1 and 2 lineages. *Nat Immunol* **6**, 1123-1132 (2005).
12. R. He, M. K. Oyoshi, H. Jin, R. S. Geha, Epicutaneous antigen exposure induces a Th17 response that drives airway inflammation after inhalation challenge. *Proc Natl Acad Sci U S A* **104**, 15817-15822 (2007).
13. I. N. Marinova, H. H. Wandall, S. Dabelsteen, Protocol for CRISPR-Cas9 modification of glycosylation in 3D organotypic skin models. *STAR protocols* **2**, 100668 (2021).
14. M. Chen *et al.*, TMEM79/MATTRIN defines a pathway for Frizzled regulation and is required for Xenopus embryogenesis. *Elife* **9**, e56793 (2020).
15. H. Hennings *et al.*, Calcium regulation of growth and differentiation of mouse epidermal cells in culture. *Cell* **19**, 245-254 (1980).
16. Y. Poumay, M. R. Pittelkow, Cell density and culture factors regulate keratinocyte commitment to differentiation and expression of suprabasal K1/K10 keratins. *Journal of Investigative Dermatology* **104**, 271-276 (1995).
17. J. P. Smits *et al.*, Immortalized N/TERT keratinocytes as an alternative cell source in 3D human epidermal models. *Scientific reports* **7**, 11838 (2017).
18. A. Dobin *et al.*, STAR: ultrafast universal RNA-seq aligner. *Bioinformatics* **29**, 15-21 (2013).
19. M. Ashburner *et al.*, Gene ontology: tool for the unification of biology. *Nature genetics* **25**, 25-29 (2000).
20. E. Y. Chen *et al.*, Enrichr: interactive and collaborative HTML5 gene list enrichment analysis tool. *BMC bioinformatics* **14**, 128 (2013).
21. M. V. Kuleshov *et al.*, Enrichr: a comprehensive gene set enrichment analysis web server 2016 update. *Nucleic acids research* **44**, W90-W97 (2016).
22. A. Liberzon *et al.*, The molecular signatures database hallmark gene set collection. *Cell systems* **1**, 417-425 (2015).
23. A. Subramanian *et al.*, Gene set enrichment analysis: a knowledge-based approach for interpreting genome-wide expression profiles. *Proceedings of the National Academy of Sciences* **102**, 15545-15550 (2005).
24. S. M. Smith, M. B. Wunder, D. A. Norris, Y. G. Shellman, A simple protocol for using a LDH-based cytotoxicity assay to assess the effects of death and growth inhibition at the same time. *PloS one* **6**, e26908 (2011).
25. E. Pap *et al.*, Ratio-fluorescence microscopy of lipid oxidation in living cells using C11-BODIPY581/591. *FEBS letters* **453**, 278-282 (1999).
26. G. P. Drummen, L. C. van Liebergen, J. A. O. den Kamp, J. A. Post, C11-BODIPY581/591, an oxidation-sensitive fluorescent lipid peroxidation probe:(micro) spectroscopic characterization and validation of methodology. *Free Radical Biology and Medicine* **33**, 473-490 (2002).
27. N. A. Franken, H. M. Rodermond, J. Stap, J. Haveman, C. Van Bree, Clonogenic assay of cells in vitro. *Nature protocols* **1**, 2315-2319 (2006).
